# Granzyme B cleaves tenascin-C to release its C-terminal domain in rheumatoid arthritis

**DOI:** 10.1172/jci.insight.181935

**Published:** 2024-10-30

**Authors:** Alexandre Aubert, Amy Liu, Martin Kao, Jenna Goeres, Katlyn C. Richardson, Lorenz Nierves, Karen Jung, Layla Nabai, Hongyan Zhao, Gertraud Orend, Roman Krawetz, Philipp F. Lange, Alastair Younger, Jonathan Chan, David J. Granville

**Affiliations:** 1 International Collaboration on Repair Discoveries (ICORD) Centre, British Columbia Professional Firefighters’ Burn and Wound Healing Group, Vancouver Coastal Health Research Institute, and; 2 Department of Pathology and Laboratory Medicine, University of British Columbia, Vancouver, British Columbia, Canada.; 3 Michael Cuccione Childhood Cancer Research Program and the BC Children’s Hospital Research Institute, Vancouver, British Columbia, Canada.; 4 The Tumor Microenvironment Laboratory, INSERM U1109, Hôpital Civil, Institut d’Hématologie et d’Immunologie, Fédération de Médecine Translationnelle de Strasbourg, Strasbourg, France.; 5 McCaig Institute for Bone and Joint Health, Cell Biology and Anatomy, Cumming School of Medicine, University of Calgary, Calgary, Alberta, Canada.; 6 Department of Orthopaedics, Foot & Ankle Research, St. Paul’s Hospital, Vancouver, British Columbia, Canada.; 7 Department of Medicine, Division of Rheumatology, University of British Columbia, Vancouver, British Columbia, Canada.; 8 Arthritis Research Canada, Vancouver, British Columbia, Canada.

**Keywords:** Autoimmunity, Inflammation, Extracellular matrix, Proteases, Rheumatology

## Abstract

Rheumatoid arthritis (RA) is a common autoimmune disorder characterized by exacerbated joint inflammation. Despite the well-documented accumulation of the serine protease granzyme B (GzmB) in RA patient biospecimens, little is understood pertaining to its role in pathobiology. In the present study, tenascin-C (TNC) — a large, pro-inflammatory extracellular matrix glycoprotein — was identified as a substrate for GzmB in RA. GzmB cleaves TNC to generate 3 fragments in vitro: a 130 kDa fragment that remains anchored to the matrix and 2 solubilized fragments of 70 and 30 kDa. Mass spectrometry results suggested that the 30 kDa fragment contained the pro-inflammatory TNC C-terminal fibrinogen-like domain. In the synovial fluids of patients with RA, soluble levels of GzmB and TNC were significantly elevated compared with healthy controls. Further, immunoblotting revealed soluble 70 and 30 kDa TNC fragments in the synovial fluids of patients with RA, matching TNC fragment sizes generated by GzmB cleavage in vitro. Granzyme K (GzmK), another serine protease of the granzyme family, also cleaves TNC in vitro; however, the molecular weights of GzmK-generated TNC fragments did not correspond to TNC fragment sizes detected in patients. Our data support that GzmB, but not GzmK, contributes to RA through the cleavage of TNC.

## Abstract 2

GzmB is the protease responsible for the cleavage of Tenascin-C, an extracellular matrix molecule and an endogenous trigger of inflammation, in patients with rheumatoid arthritis

## Introduction

Granzyme B (GzmB) is a serine protease extensively studied for its role in cytotoxicity (1). Facilitated by the pore-forming protein perforin, GzmB is delivered into the cytoplasm of targeted cells where it cleaves intracellular substrates to promote caspase-dependent and caspase-independent apoptosis (1). However, the restricted view of GzmB as a mere mediator of cell death has been challenged as extracellular functions for the protease have been identified.

While approximately one-third of GzmB may leak from the immunological synapse during target cell engagement (2, 3), GzmB is also secreted into the extracellular space by immune cells that neither express perforin nor form immunological synapses (reviewed in ref. 4). Consequently, and in contrast with its minimal to undetectable levels in healthy individuals, extracellular accumulation of GzmB is observed in several pro-inflammatory conditions such as autoimmune blistering diseases (5, 6), allergic and chronic airway disease (7), cardiovascular diseases (8), or inflammatory bowel disorders (9). Due to the absence of an endogenous extracellular inhibitor in humans (10, 11), GzmB retains its proteolytic activity in the extracellular space and can contribute to disease progression by cleaving extracellular matrix (ECM) molecules. GzmB notably cleaves fibrillin-1 (8), fibronectin (12), and decorin (13), leading to collagen disorganization, impaired ECM remodeling, as well as release and subsequent activation of matrix-bound VEGF (12) and TGF-β (13). GzmB also cleaves the matricellular protein (MCP) thrombospondin-1 in age-related macular degeneration (14).

In recent years, GzmB has been detected in rheumatoid arthritis (RA) as well as other rheumatic disorders and hypothesized to mediate disease pathogenesis (reviewed in ref. 15). Extracellular GzmB is elevated in the plasma and synovial fluid (SF) of patients with RA (16). Additionally, GzmB serum levels correlate with RA severity (17) as well as the presence of radiographic erosions in Rheumatoid factor^+^ patients (18). In RA lesions, GzmB is expressed by CD8^+^ T cells (19, 20), NK/NKT cells (21), macrophages (22), CD19^+^ B cells (23), chondrocytes (24), and synovial cells (25). Expansion of *GZMB*-expressing CD8^+^ T cells is also observed in seropositive RA patients harboring anti-citrullinated protein antibodies (20).

Despite several reports documenting elevated GzmB in RA, its role remains poorly understood. In addition to its ability to promote chondrocyte apoptosis (26), GzmB directly contributes to cartilage degradation though the cleavage of aggrecan (27, 28). Additionally, while GzmB has been proposed as a potential mediator of autoantigen generation (29) with several of its substrates identified as putative autoantigens in autoimmune disorders (30), peptidylaginine deiminase 4 is the only GzmB substrate validated as an autoantigen in RA (31). Consequently, further investigation is required to elucidate the specific impact of GzmB on the development and progression of RA.

Tenascin-C (TNC) is a large hexametric glycoprotein of the ECM and one of the original members of the MCP group, defined as ECM proteins that do not play a primary structural function in the connective tissue (32). With prominent roles in embryonic development (33) through its ability to promote a low-adhesion state suitable for developmental cell migration (34), TNC is mainly absent in adult tissues but is elevated in SF (35) as well as serum and plasma (36) of patients with RA (37). TNC plays a dual pathogenic role in RA lesions. First, its C-terminal fibrinogen like domain (hereafter called FBG-C) can stimulate TLR-4–dependent pro-inflammatory cytokine production by macrophages and cells from the synovial membrane (38). Consequently, FBG-C intra-articular injection in mice is sufficient to promote local joint inflammation recapitulating some of the hallmarks of RA (38). Second, citrullinated TNC fragments serve as autoantigens in RA and are associated with the presence of cyclic citrullinated peptides in seropositive patients (39). Nevertheless, the mechanism involved in the release of TNC fragments into the SF of patients is poorly understood.

Herein, we postulated that TNC may be a GzmB substrate in the context of RA. In silico prediction identified the presence of several potential cleavage sites for GzmB in the TNC amino acid sequence. Using 2 sources of TNC, GzmB cleavage was verified in vitro, and 3 TNC fragments of distinct molecular size were identified, consistent with those predicted in silico. GzmB cleavage of TNC impaired its ability to act as a culture substratum, resulting in reduced fibroblast spreading and survival. Precise examination of the GzmB-generated TNC peptides by mass spectrometry identified that one of the fragments contains its pro-inflammatory fibrinogen-like domain. In patients with RA, soluble extracellular GzmB and TNC levels are positively correlated and are significantly elevated in the SF compared with healthy controls. Western blot analysis further revealed the presence of 2 TNC fragments in the SF of patients with RA, consistent with the in silico prediction and in vitro digestion. Additionally, we found that granzyme K (GzmK), another granzyme with tryptase-like activity and pro-inflammatory potential, also cleaves TNC in vitro. Nevertheless, in addition to the inconsistency between the TNC fragments generated by GzmK in vitro with those detected in patients, levels of GzmK and TNC in the SF are not correlated.

Together, our results indicate that GzmB cleaves TNC to generate fragments that are detected in the SF of patients with RA, providing additional mechanisms whereby GzmB may contribute to RA pathogenesis.

## Results

### In silico prediction identifies TNC as a GzmB substrate.

**Figure 1:**
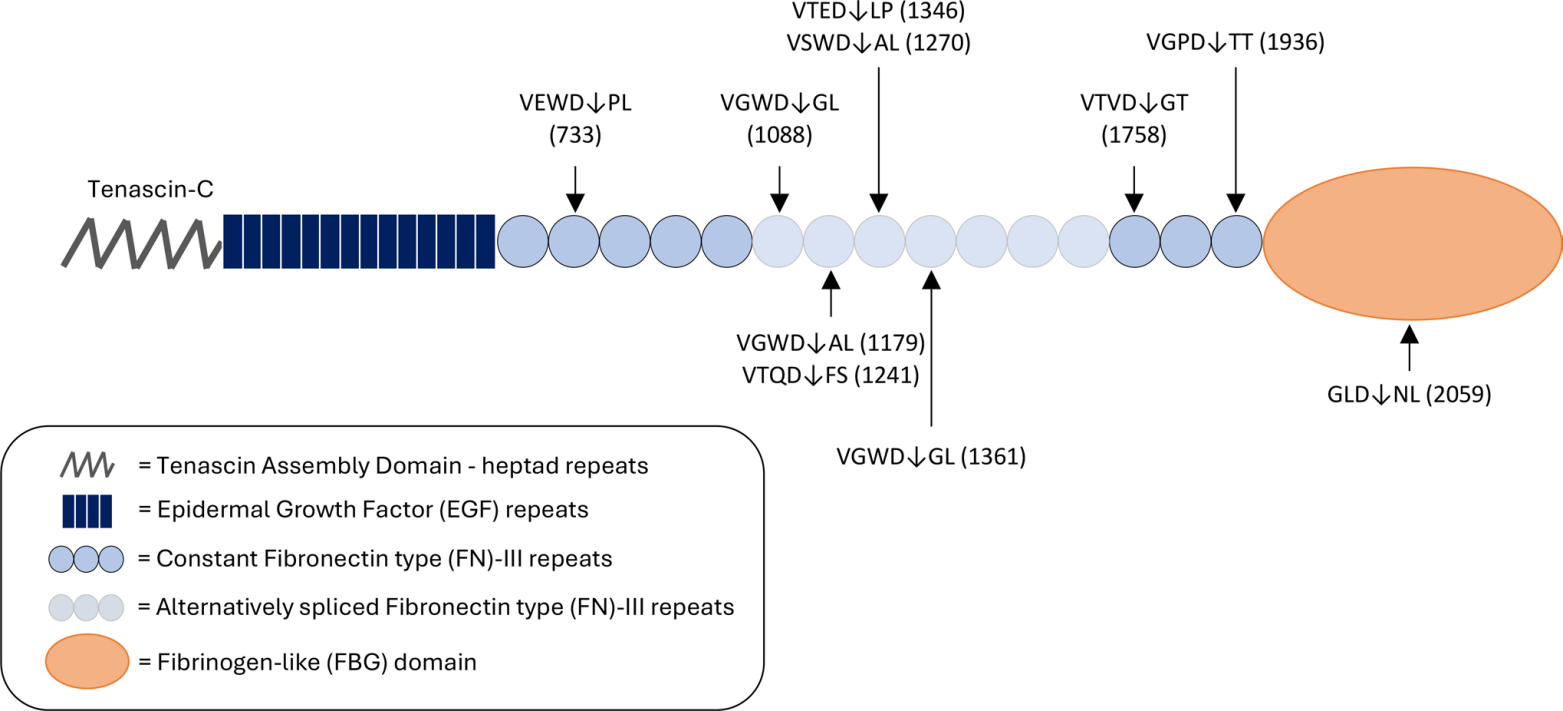
In silico prediction identifies TNC as a potentially novel GzmB substrate. Schematic diagram of predicted granzyme B (GzmB) cleavage sites on human TNC (hTNC). Letters represent the single–amino acid code of the tetrapeptide preceding as well as the dipeptide following the expected cleavage site. Arrows represent the location of the expected cleavage site (after aspartic acid, D) and correspond to the amino acid location referenced between brackets.

The amino acid sequence of human TNC (canonical sequence according to the UniProt accession no. P24821-1) was analyzed using GraBCas (40), a software that successfully predicted GzmB cleavage sites on the MCPs, thrombospondin-1 and -2 (14). In silico prediction (Table 1) identified 10 potential GzmB cleavage sites on TNC: 9 in its fibronectin type-III (FN-III) repeats (constant and alternatively spliced) as well as 1 in its C-terminal FBG-C (Figure 1). Among these, VGPD↓TT (cleavage site in position 1,936, FN-III_15_) and VTQD↓FS (cleavage site in position 1,241, FN-III_7_) were the 2 most probable cleavage sites as suggested by the cleavage scores (23.85 and 1.55, respectively; Table 1). No cleavage sites were predicted in the TNC N-terminal assembly domain (TAD/heptad repeats) or EGF repeats (Figure 1).

### GzmB cleaves TNC in vitro.

**Figure 2:**
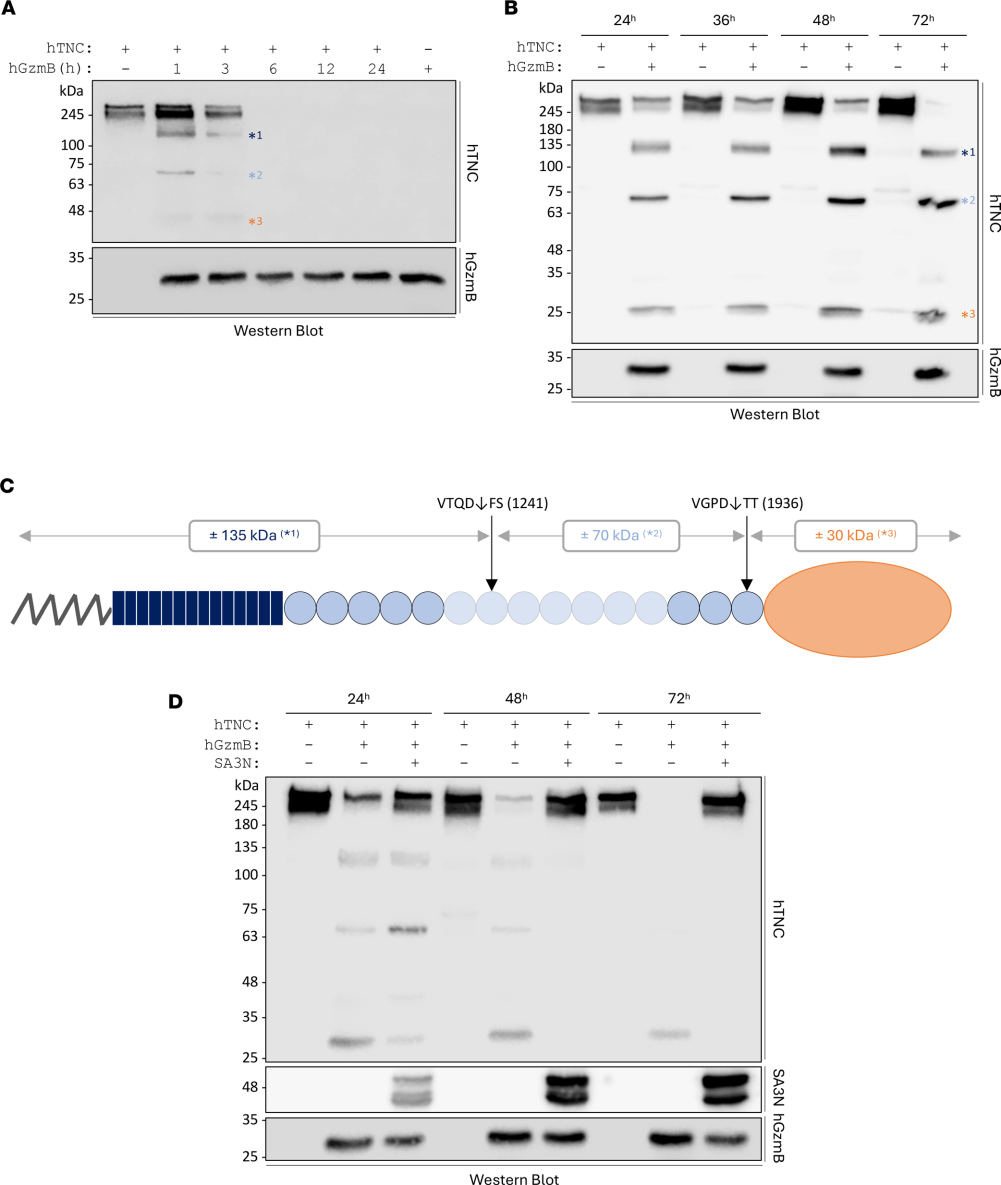
GzmB cleaves TNC in vitro. A total of 500 ng of commercial (**A**) or lab-generated (**B**) rhTNC was incubated with (or without) 50 nM rhGzmB for several time points at 37°C and analyzed by Western blot probing for TNC (upper panel) and GzmB (lower panel). GzmB generates 3 TNC peptides of distinct molecular weights, identified as *^1^, *^2^, and *^3^. (**C**) Expected repartition of the effective cleavage sites for GzmB in TNC based on the in silico prediction and the in vitro digestion. (**D**) A total of 50 nM of rhGzmB was preincubated at 37°C for 1 hour with 300 nM of SA3N before the addition of rhTNC (500 ng). Digestions were performed for 24, 48, and 72 hours followed by Western blot analysis probing for TNC, SA3N, and GzmB. Images shown are representative of at least 3 independently repeated experiments.

To investigate the potential cleavage of TNC by GzmB, cell-free in vitro digestion assays were performed using 2 sources of recombinant human (rh) TNC — a commercially available one (MilliporeSigma – Figure 2A) and one laboratory produced and purified (Figure 2B, hereafter referred to as “lab-generated”). Both sources of rhTNC were incubated with rhGzmB for different time points, followed by analysis of the degradation products by Western blotting. Similar degradation patterns were observed using the 2 sources of TNC (Figure 2, A and B). However, the commercial source of TNC was more sensitive to GzmB cleavage, with complete digestion observed between 3 hours and 6 hours (Figure 2A) compared with 72 hours for the lab-generated TNC (Figure 2B). GzmB cleavage of rhTNC generates 3 fragments of distinct molecular weight — one around 120/130 (*^1^), a second close to 70 kDa (*^2^), and a third low–molecular weight fragment around 25/30 kDa (*^3^). Generation of the 3 fragments is consistent with the simultaneous digestion of TNC by GzmB at the 2 most probable cleavage sites identified by in silico prediction, i.e., VGPD↓TT in position 1,936 and VTQD↓FS in position 1,241 (Figure 2C and Table 1). Consequently, the low–molecular weight fragment generated by GzmB may contain TNC FBG-C (Figure 2C).

Treatment with SerpinA3N (SA3N), an endogenous murine serine protease inhibitor identified as an extracellular inhibitor of human and murine GzmB (41), inhibited GzmB cleavage of rhTNC (Figure 2D). Additionally, N-deglycosylation of TNC rendered the glycoprotein more sensitive to GzmB-mediated proteolysis, with complete digestion observed for the deglycosylated lab-generated rhTNC after 24 hours (Supplemental Figure 1; supplemental material available online with this article; https://doi.org/10.1172/jci.insight.181935DS1), compared with 72 hours for the glycosylated form (Figure 2B). This finding suggests that the glycosylation pattern of TNC may modulate its susceptibility to protease degradation.

Based on the high amino acid sequence identity (67.76%, Supplemental Figure 2A) as well as the conservation of the catalytic triad position (His^59^-Asp^103^-Ser^198^; Supplemental Figure 2B) between human and mouse GzmB, we investigated the ability of mouse GzmB to cleave mouse TNC. To this end, we produced and purified recombinant murine (rm) TNC and performed in vitro cell-free digestion assay. Western blot analysis of the digestion products revealed that similar to its human counterpart, rmGzmB cleaves rmTNC in vitro in a time-dependent manner (Supplemental Figure 2C). Although the molecular weights of the generated rmTNC fragments are different from those observed using human proteins, rmGzmB also cleaves rmTNC to generate a fragment around 30 kDa (arrowheads, Supplemental Figure 2C).

Together, GzmB cleaves TNC in vitro to generate 3 fragments of distinct molecular sizes. The low–molecular weight fragment generated by GzmB digestion is predicted to contain the C-terminal FBG-C, a domain previously identified as an endogenous trigger of inflammation (38) and a citrullinated autoantigen in patients with RA (39).

### GzmB cleaves a TNC substratum to impair fibroblast spreading and viability.

**Figure 3:**
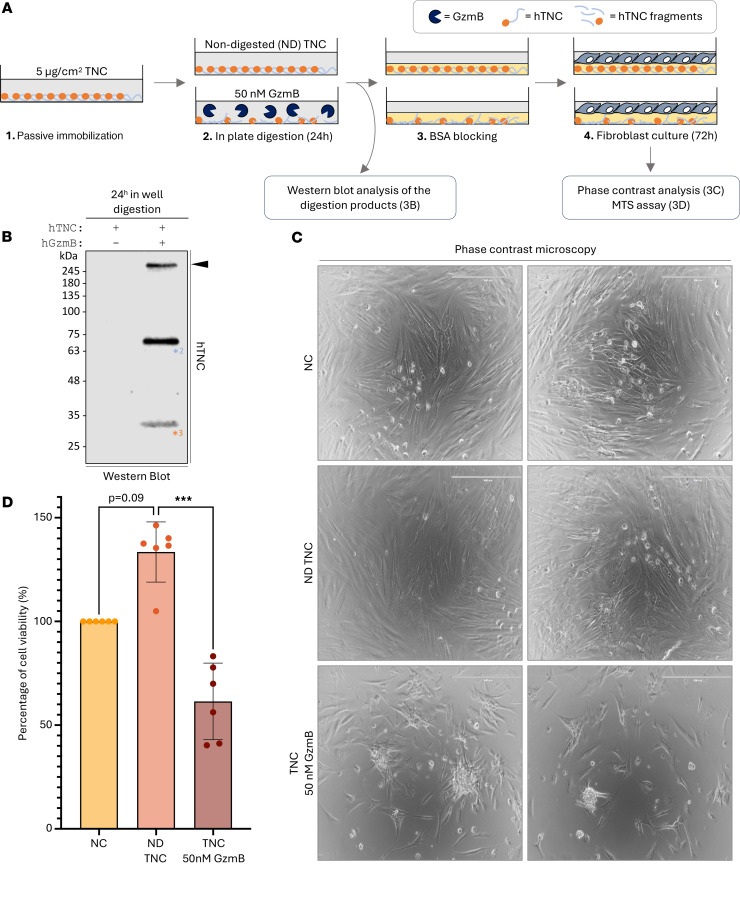
GzmB cleaves TNC substratum to impair fibroblast spreading and viability. (**A**) Schematic representation of the experimental procedure used to investigate how GzmB digestion of a TNC substratum impacts fibroblast behaviors. (**B**) Western blot analysis of the presence of soluble TNC fragments after 24-hour digestion of the immobilized TNC substratum as indicated in **A**. (**C**) Phase contrast microscopy performed on primary human dermal fibroblasts cultured for 72 hours onto noncoated (NC) wells, wells coated with nondigested (ND) rhTNC, or wells coated with rhTNC and digested with 50 nM rhGzmB as indicated in **A**. Bars, 400 μm. (**D**) Percentage of cell viability obtained by MTS assay performed on primary human dermal fibroblasts cultured for 72 hours onto NC wells, wells coated with ND rhTNC, or wells coated with rhTNC and digested with 50 nM rhGzmB as indicated in **A**. Results were normalized compared to the NC condition and are represented as means ± SD from 6 independent experiments. Statistical analyses were performed using nonparametric ANOVA (Kruskal-Wallis rank-sum test) followed by Dunn’s multiple comparisons test with Bonferroni’s *P* value adjustment. ***=*P* < 0.001. Images shown are representative of at least 3 independently repeated experiments.

Next, the impact of GzmB cleavage of TNC on cellular adhesion (34) and proliferation (42) was assessed using TNC as a culture substratum (Figure 3A). Western blot analysis of the digestion products revealed that GzmB cleaves TNC even when immobilized as a culture substratum (Figure 3B), further evidenced by reduced TNC detection at the bottom of the wells by immunofluorescence (Supplemental Figure 3). GzmB proteolysis detached some of the full-length glycoprotein (arrowhead) and generated 2 soluble fragments of 70 kDa (^*2^) and 30 kDa (^*3^) (Figure 3B) nearly identical to those previously identified by in vitro cleavage (Figure 2B). Absence of the 130 kDa fragment (referenced as ^*1^ in Figure 2) suggests that this TNC peptide remains attached to the wells (Figure 3B). Consequently, and based on our in silico prediction (Figure 2C), GzmB cleaved the immobilized TNC to generate a culture substratum most probably composed of its N-terminal fragment, encompassing the tenascin assembly domain, EGF repeats, as well as several FN-III domains.

Phase contrast analysis revealed no clear differences in the morphology of primary human fibroblasts when cultured for 72 hours onto wells coated with nondigested TNC (ND TNC) compared with noncoated (NC) wells (Figure 3C). In contrast, cells cultured onto TNC cleaved by GzmB appeared less elongated than those cultured onto control wells, indicating a potential impairment of cellular adhesion or spreading by the remaining TNC substratum. A clear reduction of cell density was also noticeable for wells coated with the digested glycoprotein (Figure 3C). MTS assay verified this macroscopic observation and revealed a reduction of fibroblast viability when cultured on the cleaved TNC substratum compared with ND TNC (Figure 3D). Despite a trend in the increased number of live cells in presence of ND TNC (*P* = 0.09), no significant differences were observed compared to the NC conditions (Figure 3D). In the absence of TNC (Supplemental Figure 4A), GzmB demonstrated no direct impact on fibroblast adhesion/morphology (Supplemental Figure 4B) or viability (Supplemental Figure 4C).

To address the potential translation of these findings to other cell types, we also assessed keratinocytes (HaCaTs) and lung epithelial cells (Beas2B). When cultured in the presence of the cleaved TNC substratum, a reduction of keratinocyte adherence (Supplemental Figure 5A, left panel) as well as the appearance of fibroblast-like features in lung epithelial cells (Supplemental Figure 5A, right panel) were observed. While keratinocyte cell viability was unchanged compared to the control conditions (Supplemental Figure 5B), reduced lung epithelial cell viability was measured on the cleaved TNC substratum (Supplemental Figure 5C), similar to the one reported for fibroblasts (Figure 3D). This observation suggests that the impact of the remaining TNC substratum is cell type dependent.

Collectively, these results suggest that from the 3 TNC fragments generated by GzmB cleavage, 2 (70 and 30 kDa) are solubilized and 1 (130 kDa) remains anchored to the ECM. When used as a culture substratum, the remaining TNC 130 kDA fragment impaired cell spreading and viability.

### GzmB proteolysis of TNC releases its C-terminal FBG-like domain in vitro.

**Figure 4:**
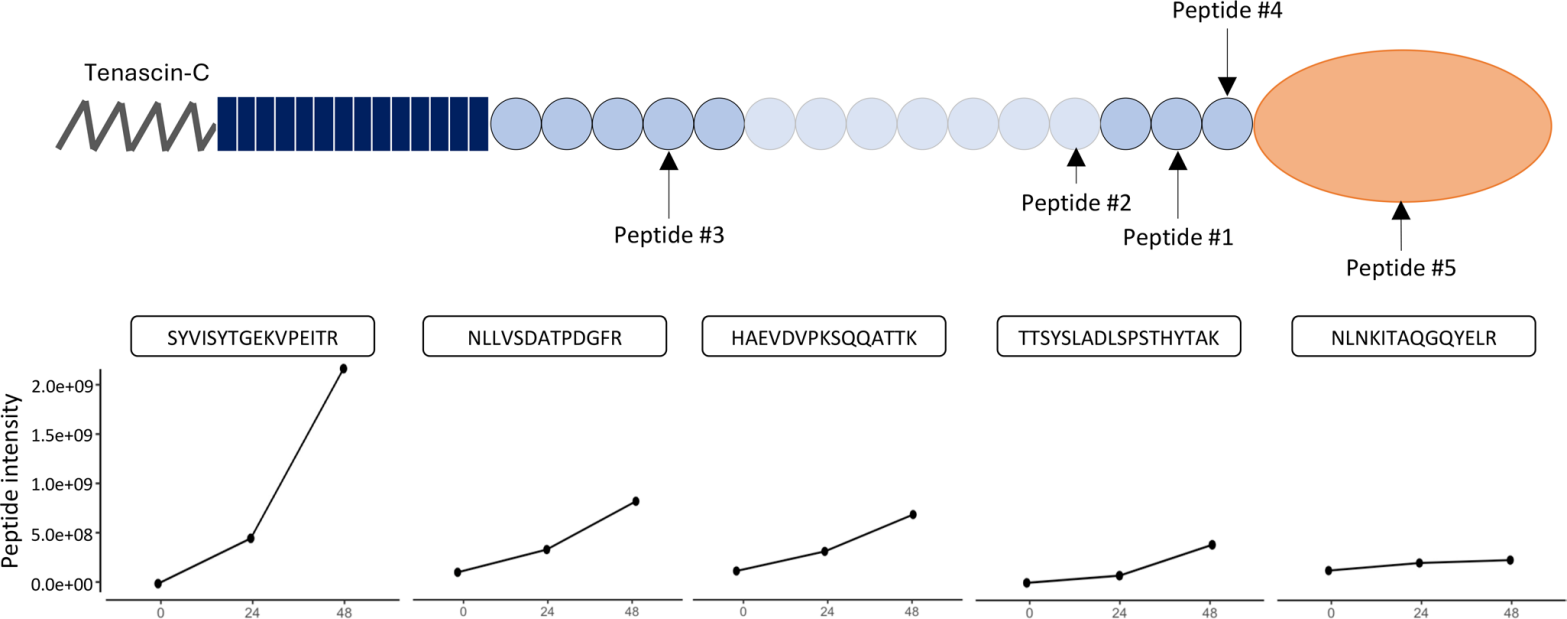
GzmB proteolysis of TNC releases its C-terminal FBG-like domain in vitro. GzmB-generated peptides from TNC were identified from the mass spectrometry data (available in Table 2) and mapped on the TNC protein. Intensity of the TNC peptide in the control, 24 hours’ digestion, or 48 hours’ digestion sample was plotted as line graphs.

Mass spectrometry analysis revealed the presence of 5 TNC N-termini generated by GzmB cell-free cleavage assay: SYVISYTGEKVPEITR (Peptide #1, cleavage site ATVD↓SY in position 1,827 — FN-III_14_), NLLVSDATPDGFR (Peptide #2, cleavage site PEVD↓NL in position 1,624 — FN-III_12_), HAEVDVPKSQQATTK (Peptide #3, SGGD↓HA in position 934 — FN-III_4_), TTSYSLADLSPSTHYTAK (Peptide #4, VGPD↓TT in position 1,936 — FN-III_15_), and NLNKITAQGQYELR (Peptide #5, LGLD↓NL in position 2,059 — FBG-C) (Figure 4 and Table 2). All TNC peptides were generated within the first 24 hours of GzmB incubation. With the exception of Peptide #5 (NLNKITAQGQYELR), intensity of the peptides increased between 24 hours and 48 hours of digestion, indicating that more of the same peptides were generated with time (Figure 4) without modification of the global repartition of peptide lengths between the 2 time points (Supplemental Figure 6).

Similar to all cleavage sites identified by in silico prediction (Figure 1), mass spectrometry analysis of the digestion products indicated that GzmB cleaves TNC mostly within its FN-III domains, with no N-termini arising from its N-terminal TAD or EGF repeats (Figure 4). The presence of Peptide #3 (HAEVDVPKSQQATTK, corresponding to SGGD↓HA cleavage in position 934) is consistent with the release of a fragment around 140 kDa and could be an explanation for the generation of the high–molecular weight fragment identified by in vitro digestion (^*1^, Figure 2). Generation of Peptides #4 and #5 by GzmB perfectly aligned with 2 cleavage sites identified by in silico prediction, VGPD↓TT (position 1,936) and LGLD↓NL (position 2,059), respectively (Table 1). Among them, VGPD↓TT was the most probable cleavage site according to the in silico prediction (score of 23.85, Table 1). Cleavage of TNC by GzmB at this specific site is consistent with the release of the 30 kDa low–molecular weight fragment identified in vitro (Figure 2B and Figure 3B), thus verifying that this fragment contains the FBG-C.

Collectively, fragment characterization is consistent with in silico prediction and in vitro cleavage results. While it verifies that the solubilized 30 kDa TNC fragment generated by GzmB contains its C-terminal FBG-like domain, it also indicates that the 130 kDa fragment — which remained attached to the ECM — is the N-terminal fragment.

### TNC fragments detected in patients with RA align with in silico prediction and in vitro digestion by GzmB.

As both GzmB and TNC levels were previously reported to be elevated in the SF of patients with RA (16, 35), GzmB-mediated cleavage of TNC was assessed in the context of RA. To do so, we collected and analyzed SF from 12 patients with RA (10F/2M, mean age 58), 7 patients with osteoarthritis (OA; 3F/4M, mean age 64), and 6 patients with non-RA inflammatory arthritis (IA; 2F/4M, mean age 41). As controls, 5 healthy SF (healthy controls, HC; cadaveric donation, 2F/3M, mean age 56) were obtained from individuals with no history of joint disease or trauma (confirmed macroscopically at the time of harvest). Individual patient information (including sex, age, disease severity, levels of C-reactive protein [CRP], seropositivity, sample type [location and collection] and past medication) is recorded in Table 3.

**Figure 5:**
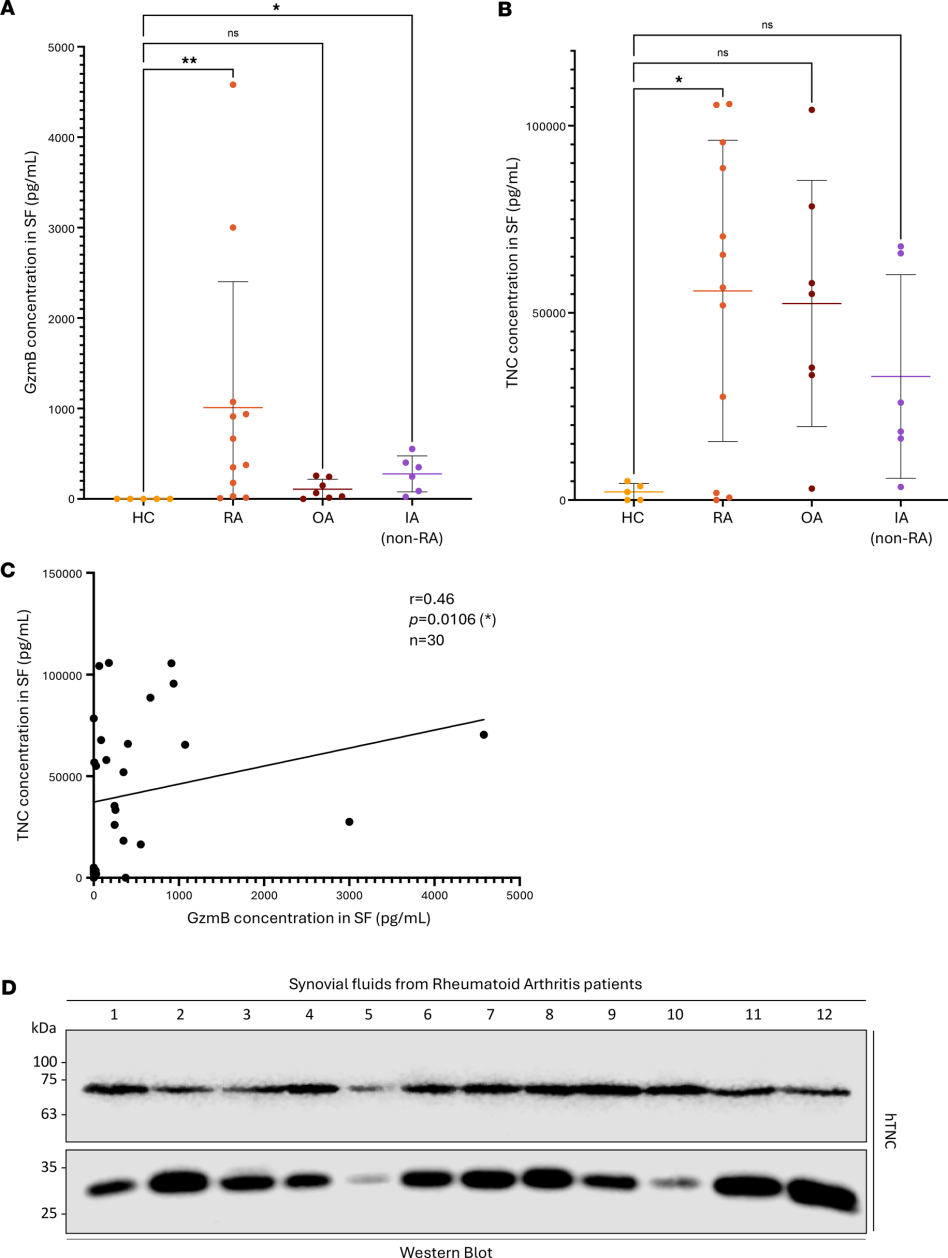
TNC fragments detected in patients with RA align with in silico prediction and in vitro digestion by GzmB. GzmB (**A**) and TNC (**B**) quantification in healthy controls (HC, *n* = 5), rheumatoid arthritis (RA, *n* = 12), osteoarthritis (OA, *n* = 7), and inflammatory arthritis (IA, *n* = 6) patient SF represented as means ± SD. Statistical analyses were performed using nonparametric ANOVA (Kruskal-Wallis rank-sum test) followed by Dunn’s multiple comparisons test with Bonferroni’s *P* value adjustment. (**C**) Correlation (Spearman rank-order) between TNC and GzmB levels in HC, RA, OA, and IA patients’ SF (*n* = 30). (**D**) Western blot analysis demonstrating the presence of 2 TNC fragments (70 kDa and 30 kDa) in the SF of 12 patients with RA. *=*P* < 0.05, **=*P* < 0.01.

GzmB (Figure 5A) and TNC (Figure 5B) levels were significantly elevated in the SF of patients with RA compared with HC. GzmB levels were also significantly elevated in the SF of patients with IA compared with HC (Figure 5A). No significant differences were observed between RA and OA, RA and IA, OA and IA, or OA and HC. A moderately positive correlation (*r* = 0.46, *P* = 0.0106) was identified between levels of soluble TNC and GzmB in our cohort of patients (Figure 5C), indicating that more TNC was detected in the SF of individuals with high levels of extracellular GzmB. Western blot analyses performed on SF from 12 patients with RA demonstrated the presence of 2 TNC fragments of approximately 70 kDa and 30 kDa (Figure 5D), matching 2 of the 3 TNC fragments generated (Figure 2B) and solubilized (Figure 3B) after GzmB cleavage in vitro.

### GzmK cleaves TNC in vitro, but the generated fragments do not match those identified in patients.

GzmK is a pro-inflammatory tryptase-like protease of the granzyme family (43). As GzmK-expressing CD8^+^ T cells are expanded in RA biospecimens (19), we investigated whether GzmK could contribute to RA through the cleavage of TNC.

**Figure 6:**
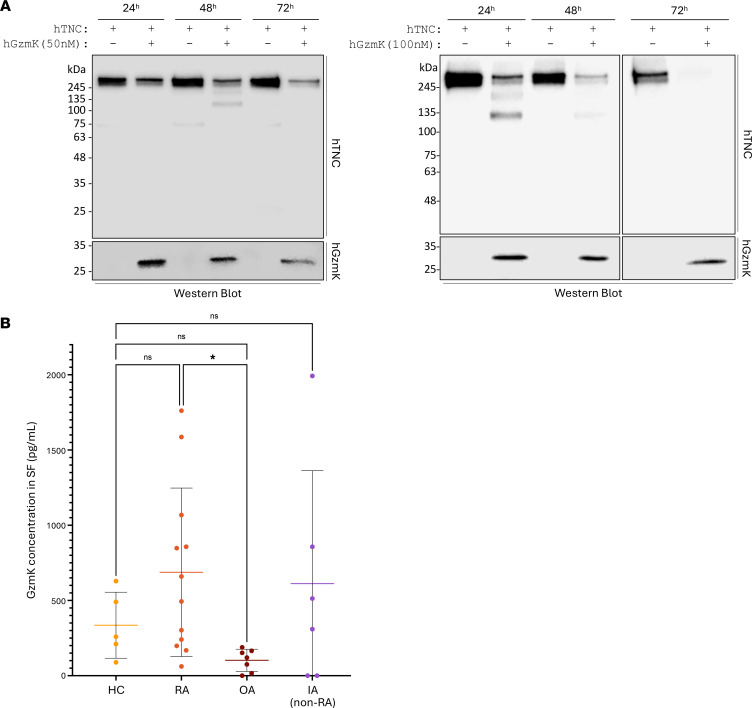
GzmK cleaves TNC in vitro, but the generated fragments do not match those identified in patients. (**A**) A total of 500 ng of lab-generated rhTNC was incubated with 50 or 100 nM of rhGzmK for 24 hours, 48 hours, or 72 hours at 37°C and analyzed by Western blot probing for TNC (upper panel) and GzmK (lower panel). Images shown are representative of at least 3 independently repeated experiments. (**B**) GzmK quantification in healthy controls (HC, *n* = 5), rheumatoid arthritis (RA, *n* = 12), osteoarthritis (OA, *n* = 7), and inflammatory arthritis (IA, *n* = 6) patient SF represented as means ± SD. Statistical analyses were performed using nonparametric ANOVA (Kruskal-Wallis rank-sum test) followed by Dunn’s multiple comparisons test with Bonferroni’s *P* value adjustment. *=*P* < 0.05.

The gradual reduction of full-length rhTNC intensity, alongside the generation of 2 TNC fragments (>100 kDa — generated at 48 hours for 50 nM and 24 hours for 100 nM), indicate that GzmK cleaved TNC in vitro in a time-dependent manner (Figure 6A). At the same concentration, GzmK cleaved TNC less efficiently than GzmB (full-length TNC still detectable after 72-hour incubation with 50 nM GzmK) and with a digestion pattern that is inconsistent with the molecular weight of TNC fragments identified in the SF of patients with RA (Figure 5D). While GzmK levels were significantly elevated in the SF of patients with RA compared with OA (but not HC, Figure 6B), no positive correlation was observed between GzmK and TNC levels (*r* = 0.11, *P* = 0.5502, Supplemental Figure 7A) in the SF of the patient cohort. A weak but statistically significant positive correlation was identified between the SF levels of GzmK and GzmB (*r* = 0.38, *P* = 0.0382, Supplemental Figure 7B).

These results indicate that GzmK is elevated in RA patient SF and can cleave TNC in vitro. Nevertheless, the absence of a correlation between GzmK and TNC levels in SF, coupled with inconsistencies between the molecular weights of generated TNC fragments in vitro and in patients, suggests that GzmK does not directly cleave TNC in patient SF.

## Discussion

Extensively studied for its intracellular pro-apoptotic properties, GzmB is detected in extracellular fluids of patients with autoimmune or chronic inflammatory conditions. This is notably the case in RA, with several reports indicating the accumulation of extracellular GzmB in patient SF, serum, and plasma. Nevertheless, the precise role of GzmB in RA remains elusive, hindering its use as a predictive tool, biomarker, or therapeutic target for the disease.

Our results suggest that TNC is cleaved by GzmB in RA. GzmB cleaves TNC in vitro to generate 3 fragments, releasing the C-terminal FBG-like domain. Two TNC fragments of corresponding molecular weights are also detectable in the SF of patients with RA, supporting the hypothesis that GzmB cleavage of TNC also occurs in vivo. As the FBG-like domain of TNC, released by GzmB, has previously demonstrated pro-inflammatory properties (38), results from this study support the contribution of GzmB to joint inflammation through the cleavage of TNC.

While increasing numbers of studies have reported the expansion of GzmK-expressing CD8^+^ T cell populations in rheumatic disorders (15), the recognized role of extracellular GzmK in RA is currently restricted to pro-inflammatory cytokine production (19). In the present study, while indeed GzmK cleaved TNC in vitro, the molecular weights of TNC fragments were not consistent with those detected in SF, suggesting that GzmK does not contribute to TNC fragmentation in RA. This might be due to GzmK extracellular inhibition by bikunin, a GzmK inhibitor endogenously present in human plasma (44, 45). Investigating the presence and levels of bikunin will be the next step to decipher whether GzmK retains its extracellular proteolytic activity in the SF of patients with RA.

In addition to its role as a local mediator of inflammation (38), TNC inhibits cellular adhesion to fibronectin, promoting a low-adhesion state suitable for developmental cell migration (33) and metastatic colonization (46). The TNC FBG-like domain also induces EMT and cytostasis by promoting latent TGF-β activation (42). Here, we reported that TNC, when used as a culture substratum, did not significantly affect primary human dermal fibroblast attachment or viability. However, compared with undigested controls, GzmB-dependent proteolysis of the TNC substratum significantly reduced fibroblast spreading and viability. Although this will require further experimental validation, GzmB-cleaved TNC may have a similar impact on RA synovial fibroblasts (RASF). As RASF possess migratory properties that can lead to the propagation of RA to nonaffected joints (47), it is possible that the cleavage of TNC by GzmB might generate a low-adhesion ECM in inflamed joints, reducing RASF proliferation while promoting migration. It is possible that EGF repeats, as well as the Matrix Regulating Motif located on TNC FN-III_3–5_ (48) and most likely present in the 130 kDa fragment still attached to the wells after GzmB digestion, may play a central role in the regulation of fibroblast behavior. The implication of EGF receptors, which can act as ligand for TNC EGF domains (49), is an open question that will be investigated in the future.

As the 70 kDa fragment of TNC is almost detectable at the same intensity in the SF of patients with RA, we cannot exclude that the release of this fragment can be mediated by other proteases. TNC may notably be cleaved by several members of the MMP family (50, 51) with established roles in RA (52, 53). GzmB may be indirectly implicated in this process, as GzmB-dependent fibronectin cleavage generates bioactive fragments that can promote MMP-1 production by dermal fibroblasts (54). Precise characterization of TNC fragments in patient biospecimens will be the next step to confirm the involvement of GzmB in the generation of TNC fragments in RA. Characterization of these fragments will identify TNC isoform(s) expressed in RA patient lesions and provide more information regarding their posttranslational modification profiles, 2 parameters that can modulate TNC activity (55) and susceptibility to extracellular processing.

The TNC C-terminal FBG-like domain has been previously linked to RA (38). Our results suggest that GzmB promotes the release of this domain into the SF of patients with RA. In fact, TNC FBG-like domain is citrullinated, and its levels correlate with the presence of cyclic citrullinated peptide in patients with RA (39). FBG-C can also stimulate a TLR-4/MyD88-dependent signaling pathway in primary human macrophages and synovial fibroblasts, leading to their production and secretion of pro-inflammatory cytokines (38). Additionally, intra-articular injection of purified recombinant FBG-C in vivo into mice is sufficient to induce local joint inflammation that recapitulates several hallmarks of RA (38). Consequently, by releasing this specific TNC domain, GzmB may promote the onset and progression of RA. Further studies are required to confirm the antigenicity of GzmB-generated fragments. A deeper analysis of the cell source of GzmB will be also an important step in understanding its precise implication in RA, as a protective role for CD19^+^ B cell–derived GzmB has been previously reported (23).

In addition to being a promising biomarker for joint inflammation, increased attention has been recently forwarded pertaining to the use of TNC as a potential therapeutic approach for rheumatic diseases. For instance, VIB4920 (dazodalibep) is a fusion protein composed of the third TNC FN-III domain that can act as an antagonist for human CD40 ligand (56). VIB4920 is currently under clinical investigation for RA. In a phase II clinical trial performed on 78 patients with RA (MIDORA; NCT04163991), VIB4920 demonstrated an acceptable safety profile with a significant reduction of DAS28-CRP compared with the placebo group (57), confirming the relevance of using a TNC-based approach for the treatment of RA.

It is possible that treatments (e.g., DMARD), as well as sampling procedure (e.g., flare-ups versus arthroplasties) may influence the levels of GzmB and TNC measured in SF. For example, 3 patients with RA (RA3, RA4, and RA5) as well as 2 patients with IA (IA3 and IA5) were treated with upadacitinib (RINVOQ), a JAK inhibitor commonly used to manage different subtypes of IA. As GzmB expression is promoted by the JAK/STAT signaling pathway (58) and reduced in mice treated with the JAK/STAT inhibitor tofacitinib (59), this may explain the discrepancies in GzmB levels measured in the SF of patients with IA (RA and non-RA). With regard to the low levels of TNC measured in patients RA5, RA9, RA10, and RA11, SF were collected during ankle arthroplasties in the absence of arthritic flare. Thus, we hypothesize that the release of TNC fragments from the synovium is not passively induced by GzmB but promoted during excessive inflammation as observed during arthritic flares.

Considering the extracellular functions of granzymes in numerous pro-inflammatory disorders, our data depicting GzmB cleavage of TNC shed light on the potential contribution of the protease to RA, thus supporting its use as a promising therapeutic target.

## Methods

### Sex as a biological variable

Both sexes (female and male) were involved in this study, and the female/male ratio was reported for each group. Based on the size of our cohort, female and male biospecimens were analyzed together without sex dissociation.

### Prediction of GzmB cleavage sites in human TNC

The protein sequence of human TNC was obtained from UniProt (accession no.: P24821-1) and assessed for potential GzmB cleavage sites using GraBCas (v1.0) software with a cutoff score of 0.1 (40). Identified sites were mapped to their respective regions in the full-length glycoprotein according to the domain organization available on UniProt. Predicted molecular size of the predicted cleavage products was estimated using the Compute pI/Mw tool from ExPASy (https://web.expasy.org/compute_pi/).

### Recombinant TNC production and purification

Human embryonic kidney 293 (HEK293) cells expressing Epstein-Barr Nuclear Antigen 1 (EBNA1) and stably transfected with pCEP4-BM40-6HIS/Puromycin encoding full-length human hTNC were donated by G. Orend (U1109 INSERM, Strasbourg, France). HEK293 EBNA1 cells stably transfected with pCEP-6HIS/Puromycin encoding murine (m)TNC (GenBank: D90343.1, cloned as previously described for hTNC in ref. 60) were donated by Ruth Chiquet-Ehrismann (Friedrich Miescher Institute for Biomedical Research, Basel, Switzerland). Both cell lines were maintained in complete DMEM containing 10% (v/v) fetal bovine serum (Gibco) and 1% (v/v) Penicillin-Streptomycin (MilliporeSigma) and selected with 2.5 μg/mL of Puromycin (Gibco). For protein production, stably transfected HEK293 cells were cultured to overconfluence for 3 weeks under serum deprivation, and culture medium was collected 3 times a week. Conditioned media enriched in TNC were cleared from cellular debris by centrifugation at 300*g* for 10 minutes at room temperature and stored at –20°C.

Full-length hTNC and mTNC were purified as described by Giblin et al. 2018 (61). Before purification, histidine-tagged TNC was precipitated from conditioned medium by stirring for 2 hours in ice in the presence of 2.2 M ammonium sulfate (Fisher Chemical, Thermo Fisher Scientific), pelleted by centrifugation at 12,000*g* for 20 minutes at 4°C, resuspended in 50 mL of phosphate-buffered saline (PBS, MilliporeSigma) containing 0.01% Tween-20 (MilliporeSigma), and dialyzed 3 times (twice for 2 hours, then overnight) at 4°C in PBS containing 0.01% Tween-20 using 6–8 kDa dialysis membrane (SpectrumLabs). As no residual fibronectin was detected by Western blot, the precipitated TNC resuspended in PBS was directly applied on HisTrap HP His tag protein purification columns (5 mL, Cytiva Life Sciences) using the ÄKTA Start Chromatography System (GE Healthcare, now Cytiva Life Sciences). Column was washed using PBS containing 20 mM imidazole, and TNC was eluted using 300 mM imidazole diluted in PBS. Elution was fractioned by 0.5 mL using the Frac30 Fraction Collector System (GE Healthcare) and analyzed by Brilliant Blue Staining (MilliporeSigma). Fractions enriched in TNC were pooled together and dialyzed 3 times overnight at 4°C in PBS, PBS containing 0.2% (v/v) chloroform (Fisher Chemical, Thermo Fisher Scientific), and PBS before storage at –80°C. TNC concentration was determined using the Pierce BCA Protein Assay Kit (Thermo Fisher Scientific).

### In vitro cell-free cleavage assay of recombinant TNC by GzmB and GzmK

#### GzmB digestion assay.

For hTNC, 500 ng of recombinant hTNC produced at the laboratory or purchased from a commercial source (MilliporeSigma) was digested with rhGzmB (50 nM; Emerald Biostructures) in 50 mM Tris (Fisher Bioreagents) diluted in PBS, pH 7.4, at 37°C in a water bath. For mTNC, 500 ng of rmTNC produced at the laboratory was digested with rmGzmB (50 nM; Emerald Biostructures) under the same conditions.

#### GzmK digestion assay.

A total of 500 ng of lab-generated rhTNC was digested with recombinant human GzmK (50 nM or 100 nM; Bon-Opus) in 50 mM Tris diluted in PBS, pH 7.4, at 37°C in a water bath.

Reactions were stopped by adding 6× protein loading buffer (final concentration of 1×) followed by heat denaturation at 95°C for 10 minutes. For experiments using SA3N, 300 nM of SA3N (donated by Chris Bleackley, University of Alberta, Edmonton, Alberta, Canada) was preincubated with 50 nM of rhGzmB for 1 hour at 37°C in a water bath before the addition of 500 ng of rhTNC. For Supplemental Figure 1, rhTNC was deglycosylated using PNGase F (New England Biolabs) according to the manufacturer’s recommendations. Briefly, 1 μg of rhTNC was denatured at 100°C for 10 minutes in the presence of 1× glycoprotein denaturing buffer. Then, Glycobuffer 2 (1× final) and 10% NP-40 and PNGase F were added to the solution and incubated at 37°C for 1 hour in a water bath. After deglycosylation, GzmB dilution buffer (50 mM Tris in PBS, pH 7.4) as well as 50 nM rhGzmB were added to the solution, and digestion was performed at 37°C for 24 hours. Reaction was stopped as previously described.

### Protein adsorption, phase contrast microscopy, and MTS assay

rhTNC was diluted in PBS and immobilized overnight at 4°C in 96-well, flat-bottom culture plates (SARSTEDT) at a final density of 5 μg/cm^2^. Wells were then washed with PBS and incubated with 50 mM Tris (diluted in PBS), pH 7.4, alone (ND TNC) or with 50 nM GzmB for 24 hours at 37°C. Digestion products were collected and stored at –20°C, and nonspecific interaction sites were saturated using 1% bovine serum albumin (MilliporeSigma, diluted in PBS) for 1 hour at 37°C. Wells were then washed with PBS, and 5,000 primary human fibroblasts (extracted and isolated at the laboratory) in complete DMEM, 10,000 keratinocytes (HaCaTs, RRID:CVCL_0038) in complete DMEM, or 10,000 lung epithelial cells (Beas2B, ATCC, CRL-3588) in complete DMEM:F12 (Gibco) were cultured on the remaining substratum at 37°C (5% CO_2_). Phase contrast pictures were taken after 72 hours using a digital inverted microscope (EVOS FL, Life Technologies) at 10× original magnification. To quantify cellular viability, 20 μL of CellTiter 96 AQueous One Solution Cell Proliferation Assay (Promega) was added to each well and incubated for 3 hours at 37°C. Optical density was measured at 490 nm using Infinite M1000PRO spectrophotometer (Tecan). Percentage of cell viability was calculated and normalized to the NC conditions. To assess the direct impact of rhGzmB in the impairment of fibroblast viability, the exact same experimentation was performed in the absence of immobilized TNC (Supplemental Figure 4).

### Immunoblotting

A total of 20 μg (quantified by NanoDrop 2000, Thermo Fisher Scientific) of protein from patient SF, as well as in vitro digestion products, were heat-denatured at 95°C for 10 minutes. Samples were resolved by SDS-PAGE in Tris-Glycine (TG) buffer containing SDS (Tris-base from Fisher Bioreagents, Glycine from Fisher Chemical, Thermo Fisher Scientific, SDS from Bio-Rad) before being transferred onto a PVDF membrane (0.2 μm pore size, Bio-Rad) at 0.4 A for 2 hours in TG buffer containing 10% ethanol (VWR). Membranes were blocked using 10% (w/v) nonfat dry milk diluted in Tris-buffered saline (TBS, Tris-base and NaCl from Fisher Bioreagents) containing 0.1% (v/v) Tween-20 (T-TBS) at room temperature for 1 hour prior to incubation at 4°C overnight with primary antibody (orbital shaker). Rabbit monoclonal antibody anti-TNC (diluted at 1:1,000 [in vitro digestion and 70 kDa fragments from SF] or 1:500 [30 kDa fragments from SF] in T-TBS containing 5% [w/v] nonfat dry milk) was purchased from Abcam (catalog ab108930). Mouse monoclonal antibody anti-hGzmB (diluted at 1:4,000 in T-TBS containing 5% [w/v] nonfat dry milk) was purchased from BD Biosciences (catalog 550558). Rat monoclonal antibody anti-mGzmB (diluted at 1:500 in T-TBS containing 5% [w/v] nonfat dry milk) was purchased from R&D Systems, Bio-Techne (catalog MAB1865). Rabbit monoclonal antibody anti-hGzmK (diluted at 1:2,000 in T-TBS containing 5% [w/v] nonfat dry milk) was purchased from R&D Systems, Bio-Techne (catalog MAB10216). Goat anti-mouse SA3N antibody (diluted at 1:1,000 in T-TBS containing 5% [w/v] nonfat dry milk) was purchased from R&D Systems, Bio-Techne (catalog AF4709). HRP-conjugated secondary goat anti-rabbit (Bio-Rad catalog 170-6515), goat anti-mouse (Bio-Rad catalog 170-6516), donkey anti-goat (Jackson ImmunoResearch catalog 705-035-003), and goat anti-rat (Santa Cruz Biotechnology catalog 5C2303) antibodies were diluted at 1:10,000 in T-TBS containing 10% (w/v) nonfat dry milk and incubated at room temperature for 1 hour. Signal was detected using Pierce ECL Western Blotting Substrate (Thermo Fisher Scientific) for in vitro digestion and using SuperSignal West Pico PLUS chemiluminescent substrate (Thermo Fisher Scientific) for experimentations using patient samples. Images were acquired on a LI-COR Odyssey Fc system and analyzed using the FIJI software (SciJava).

### Immunofluorescence against immobilized TNC

rhTNC was immobilized at 5 μg/cm^2^ and digested with 50 nM rhGzmB as previously described. Wells were then washed 3 times with PBS followed by 1-hour incubation at room temperature with rabbit monoclonal antibody anti-TNC diluted at 1:100 in PBS. After 3 washes with PBS, donkey anti-rabbit antibody coupled with Alexa Fluor 594 (Invitrogen, A21207) diluted at 1:500 in PBS was incubated for 1 hour at room temperature. Pictures were taken using a fluorescence inverted microscope and analyzed using the FIJI software.

### Examination of semitryptic peptides by mass spectrometry to identify putative cleavage sites

We digested 1 μg of lab-generated rhTNC with 50 nM rhGzmB for 24 hours or 48 hours in 50 mM Tris diluted in PBS, pH 7.4, at 37°C in a water bath, before inhibition for 1 hour using 300 nM SA3N as well as heat inactivation for 5 minutes at 95°C. In preparation for trypsin digestion, the pH of the samples was adjusted to approximately 8.0–8.5 using 1 μL 1.0 M HEPES, pH 8.0. For each sample, 20 ng of trypsin was added, and samples were incubated in ThermoMixer C (Eppendorf) for 3 hours at 37°C and 1,000 rpm.

After trypsin digestion, the samples were desalted and prepared for liquid chromatography-tandem mass spectrometry (LC-MS/MS) analysis using STaGE tips (3M). Each STaGE tip was composed of a P200 pipette tip fitted with 2-inch circular Solid Phase Extraction SDB-RPS disks (3M) that were created using a flat-end needle as a puncher. The STaGE tips were conditioned with the following reagents: 100 μL acetonitrile (ACN) followed by 100 μL 30% methanol (MeOH) with 1% trifluoroacetic acid (TFA), and finally, 100 μL 0.2% TFA in water. The samples were acidified with TFA (1% TFA, final concentration) before loading it into the STaGE tip. The flow-through was collected and reloaded. STaGE tips were washed with the following reagents: 100 μL 0.2% TFA in water followed by 100 μL 99% isopropyl alcohol with 1% TFA. Samples were eluted using 100 μL 5% ammonium hydroxide (NH_4_OH) with 80% ACN. The elution buffer was removed from the samples using the speed vac until approximately 1 μL was left. The samples were resuspended in 0.1% formic acid (FA) in water.

Mass spectrometric analysis was performed on a Q Exactive HF Orbitrap mass spectrometer coupled with an Easy-nLC 1200 chromatography liquid chromatography system (Thermo Fisher Scientific). Buffer A was 2% ACN and 0.1% FA. Buffer B was 95% ACN and 0.1% FA. A PepSep analytical column (25 cm × 100 μm, C18 1.9 μm, 100 Å, Bruker) was used for peptide separation. Liquid chromatography gradient was at a flow of 300 nL/min using the following 70-minute gradient profile (min:%B): 0:3, 5:9, 10:11, 15:13, 20:15, 30:17, 35:18, 45:20, 50:22, 55:24, 60:90, 70:90. Top 12 method with a full-scan mass spectrometry spectrum with a mass range of 350–1,600 *m/z* was collected at a resolution of 60,000, a maximum injection time of 50 ms, and an automatic gain control (AGC) target of 3 × 10^6^. MS/MS scan was acquired at a resolution of 15,000, a maximum injection time of 50 ms, and an AGC target of 5 × 10^4^. Normalized collision energy was set to 28. Dynamic exclusion was set to 10 seconds. Charge state exclusion was set to ignore unassigned, +1, +5, and greater charges.

Proteomics data were searched using MaxQuant (version 1.6.2.10) using a human FASTA file from UniProt (March 2020) and default settings. Enzyme and digestion types were set to trypsin/P and semispecific free N-terminus, respectively. Minimum peptide length was set to 7. Oxidation (M) and Acetyl (N-term) were set as variable modifications. Carbamidomethyl (C) was set as a fixed modification. To identify potential GzmB-generated TNC peptides, the data were filtered according to the following rules: i. Peptides that were preceded by the amino acids lysine and arginine were removed: trypsin is a protease with tryptase-like activity (cleaving after lysine or arginine), so peptides preceded by these amino acids would be more related to trypsin proteolytic activity than GzmB. ii. Peptides that were identified in control samples (i.e., not digested by GzmB) were removed. iii. Peptides that were identified in both GzmB-digested samples (i.e., present in either 24-hour or 48-hour digestion) were kept: consistently seeing a peptide in samples with GzmB provides evidence that the peptide was generated by GzmB cleavage. iv. Peptides that met the previous rules and had more than 1 peptide-spectrum matchwere prioritized: repeatedly having spectral evidence for the peptide provides stronger evidence that the peptide was generated by GzmB.

### ELISA

GzmB, GzmK, and TNC were quantified from RA (*n* = 12), OA (*n* = 7), IA (*n* = 6), and HC (*n* = 5) SF by ELISA (purchased from Invitrogen for TNC (EH446RB) and GzmB (BMS2027-2) and from Novus Biologicals, Bio-Techne, for GzmK (NBP3-11793) according to the manufacturer recommendations.

### Sequence alignment and homology calculation

Sequence alignment and identification of homology degree between human (according to the UniProt accession no. P10144), mouse (according to the UniProt accession no. P04187), rat (according to the UniProt accession no. P18291), monkey (according to the UniProt accession no. B3FWU7), pig (according to the UniProt accession no. A0A288CG12), and bovine (according to the UniProt accession no. F1MTF4) GzmB amino acid sequences has been performed using the CLUSTAL Omega software (http://www.clustal.org/omega/). Residues labeled with (*) are identical between species, residues labeled with (:) are highly conserved, and residues labeled with (.) are poorly conserved. Amino acids from the catalytic triad (His^59^-Asp^103^-Ser^198^, according to ref. 62) are identified in bold and highlighted in yellow (Supplemental Figure 2B).

### Statistics

The R software (and its graphical user interface R Commander, R Studio Team) and the Prism software (GraphPad Software) were used for graphical representations of data and statistical analyses. For GzmB, GzmK, and TNC quantification by ELISA, statistical analysis was performed using nonparametric 1-way ANOVA (Kruskal-Wallis rank-sum test) followed by Dunn’s multiple comparisons test with Bonferroni’s *P* value adjustment. Correlation coefficients *r* between GzmB and TNC, GzmK and TNC, as well as GzmB and GzmK were determined using the Spearman rank-order (nonparametric equivalent of the Pearson product-moment) correlation coefficient. *P* values less than 0.05 were considered as statistically significant and are indicated in the figure legends.

### Study approval

Use of human (female and male) biospecimens for this study was approved by the University of British Columbia, Vancouver, British Columbia, Canada (Clinical research ethics board no: H22-01990), and University of Calgary, Calgary, Alberta, Canada (Clinical research ethics board no: REB15-0005). SF from patients and HC were collected by clinical collaborators Jonathan Chan (Artus Health Center, Vancouver, British Columbia, Canada), Alastair Younger (St. Paul’s Hospital, Footbridge clinic, Vancouver, British Columbia, Canada), and Roman Krawetz (McCaig Institute for Bone and Joint Health, Calgary, Alberta, Canada). Written informed consent was obtained for each sample.

### Data availability

All supporting data values have been provided and are available in the Supporting Data Values file. Mass spectrometry data have been deposited to the Proteome Consortium (http://www.proteomeexchange.org) via the MassiVE (https://massive.ucsd.edu/) partner repository: dataset MSV000094138 (Password: grzB_collab).

## Author contributions

AA, KJ, and DJG conceived the study. AA, JG, KCR, AL, MK, and L Nabai developed methodology. AA, JG, KCR, AL, MK, and L Nierves investigated. AA visualized data. GO, RK, AY, and JC contributed reagents and human biospecimens. AA, KJ, and DJG acquired funding. L Nierves and PFL developed software. AA, KJ, HZ, and DJG contributed project administration. AA, KJ, and DJG wrote the original draft. AA, JG, KCR, AL, MK, L Nierves, L Nabai, PFL, KJ, GO, and DJG reviewed and edited the manuscript. All authors contributed to the article and approved the submitted version.

## Supplementary Material

Supplemental data

Unedited blot and gel images

Supporting data values

## Figures and Tables

**Table 1:**
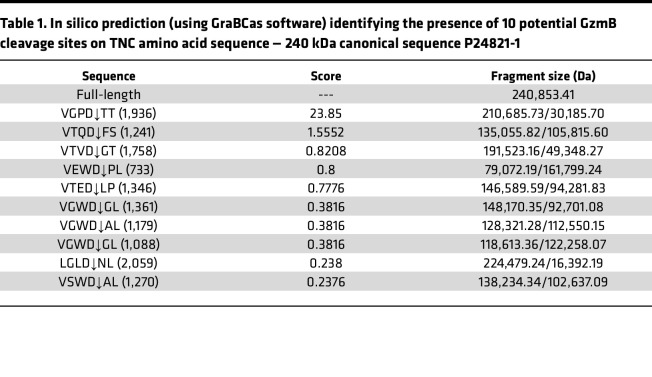
In silico prediction (using GraBCas software) identifying the presence of 10 potential GzmB cleavage sites on TNC amino acid sequence — 240 kDa canonical sequence P24821-1

**Table 2:**
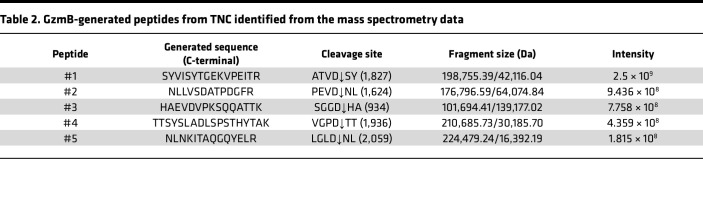
GzmB-generated peptides from TNC identified from the mass spectrometry data

**Table 3:**
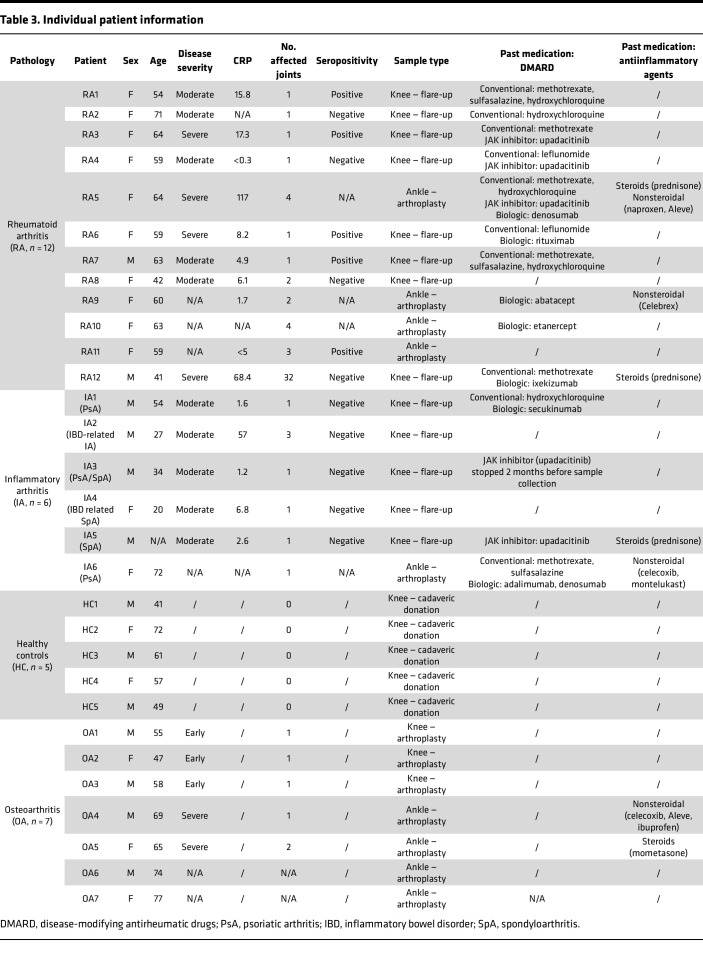
Individual patient information

## Version 1 10/30/2024

In-Press Preview

## Version 2 12/06/2024

Electronic publication
