# Supplementary material for: Granzyme B cleaves tenascin-C to release its C-terminal domain in rheumatoid arthritis
Source: JCI Insight. 2024 Oct 30;9(23):e181935. doi: 10.1172/jci.insight.181935 (PMC11623945; doi:10.1172/jci.insight.181935)
Supplement: Supplemental data [file jciinsight-9-181935-s010.pdf]

Granzyme B cleaves Tenascin-C to release its C-terminal domain in rheumatoid arthritis

Alexandre Aubert, Amy Liu, Martin Kao, Jenna Goeres, Katlyn C. Richardson, Lorenz Nierves, Karen Jung, Layla Nabai, Hongyan Zhao, Gertraud Orend, Roman Krawetz, Philipp F. Lange, Alastair Younger, Jonathan Chan, David J. Granville

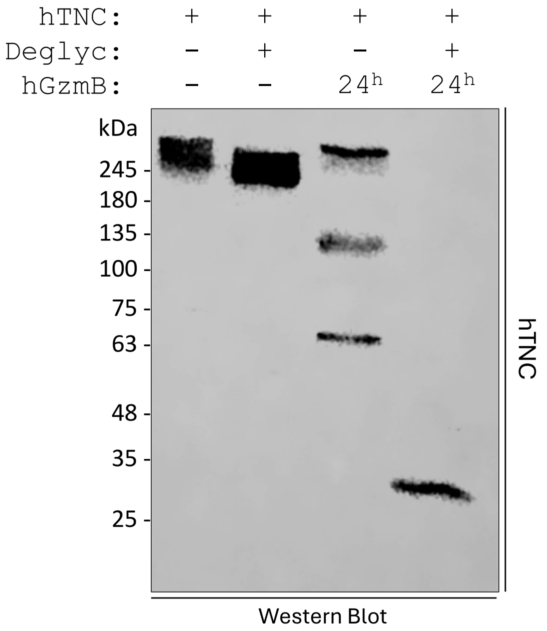

Supplementary figure 1: Western-blot analysis demonstrating that after N-deglycosylation by PNGase F, rhTNC is more sensitive to proteolytic degradation by rhGzmB.

A.

	Mouse	Rat	Human	Monkey	Pig	Bovine
**GzmB-Mouse**	/	80.57	67.76	66.94	66.94	67.62
**GzmB-Rat**	80.57	/	68.98	69.80	67.76	67.62
**GzmB-Human**	67.76	68.98	/	87.85	72.24	72.36
**GzmB-Monkey**	66.94	69.80	87.85	/	72.24	71.14
**GzmB-Pig**	66.94	67.76	72.24	72.24	/	76.73
**GzmB-Bovine**	67.62	67.62	72.36	71.14	76.73	/

B.

```
GzmB-Mouse      --MKILLLLLTLSLASRTKAGEIIGGHEVKPHSRPYMALLSIKDQQ-PEAICGGFLIRED    57
GzmB-Rat        --MKLLLLLLSFSLAPKTEAGEIIGGHEAKPHSRPYMAYLQIMDEYSGSKKCGGFLIRED    58
GzmB-Human      MQPILL--LLAFLLLPRADAGEIIGGHEAKPHSRPYMAYLMIWDQK-SLKRCGGFLIRDD    57
GzmB-Monkey     MQPILL--LLAFLLLPRTDAGEIIGGHEAKPHSRPYMAYLMIWDQM-SLKRCGGFLIRED    57
GzmB-Pig        MQPLLLLLSLAFLLPPRAEAGEIIGGHEAKPHSRPYMAYLQIQDQD-NRSRCGGFLIRED    59
GzmB-Bovine     MKPLLL--LVAFLLTPRAKAGEIIGGHEAKPHSRPYMAYLQYWNQD-VQSRCGGFLVRQD    57
                    :*   ::: *  ::.*********.********* *   ::      *****:*:*

                      His59                                       Asp103
GzmB-Mouse      FVLTAAHCEGSIINVTLGAHNIKEQEKTQQVIPMVKCIPHPDYNPKTFSNDIMLLKLKSK    117
GzmB-Rat        FVLTAAHCSGSKINVTLGAHNIKEQEKMQQIIPVVKIIPHPAYNSKTISNDIMLLKLKSK    118
GzmB-Human      FVLTAAHCWGSSINVTLGAHNIKEQEPTQQFIPVKRPIPHPAYNPKNFSNDIMLLQLERK    117
GzmB-Monkey     FVLTAAHCWGSSINVTLGAHNIKEQERTQQIIPVKRAIPHPAYNPENFSNDIMLLQLERK    117
GzmB-Pig        FVLTAAHCWGSSINVTLGAHNIKNRE-DQQVIPVRKAICHPDYNEKRISNDIMLLQLERK    118
GzmB-Bovine     FVLTAAHCNGSSIKVTLGAHNIKQQERTQQVIRVRRAISHPDYNPKNFSNDIMLLKLERK    117
                ******** ** *:*********::*  **.* : : * ** ** : :*******:*: *

GzmB-Mouse      AKRTRAVRPLNLPRRNVNVKPGDVCYVAGWGRMAPMGKYSNTLQEVELTVQKDRECESYF    177
GzmB-Rat        AKRSSAVKPLNLPRRNVKVKPGDVCYVAGWGKLGPMGKYSDTLQEVELTVQEDQKCESYL    178
GzmB-Human      AKRTRAVQPLRLPSNKAQVKPGQTCSVAGWGQTAPLGKHSHTLQEVKMTVQEDRKCESDL    177
GzmB-Monkey     AKRTTAVQPLRLPRNKAQVKPGQACDVAGWGQTTPDGKYSHTLQEVKLTVEEDQTCKSRL    177
GzmB-Pig        AKLTKAVKTLSLPGAKARVKPGQVCSVAGWGQVE-RGIYTDTLQEVKLTLQKDQECDSYL    177
GzmB-Bovine     AKQTSAVKPLSLPRAKARVKPGQTCSVAGWGRDS-TDTYADTLQEVKLIVQEDQKCEAYL    176
                ** : **: * **  :..****:.* *****:    . ::.*****:: :::*: *.: :

                                          Ser198
GzmB-Mouse      KNRYNKTNQICAGDPKTKRASFRGDSGGPLVCKKVAAGIVSYGYKDGSPPRAFTKVSSFL    237
GzmB-Rat        KNYFDKANEICAGDPKIKRASFRGDSGGPLVCKKVAAGIVSYGQNDGSTPRAFTKVSTFL    238
GzmB-Human      RHYYDSTIELCVGDPEIKKTSFKGDSGGPLVCNKVAQGIVSYGRNNGMPPRACTKVSSFV    237
GzmB-Monkey     GHYYDSTVELCVGDPEIQKASFKGDSGGPLVCNKVAQGIVSYGQRNGKPPRVCTKVSSFV    237
GzmB-Pig        PNYYNGNTQLCVGDPKKKQATFKGDSGGPLVCNNVAQGIVSYGKKDGTPPRACTKVSSFL    237
GzmB-Bovine     RNFYNRAIQLCVGDPKTKKASFQGDSGGPLVCDNVAQGIVSYGKRDGSTPRAFTKVSSFL    236
                 : ::   ::*.***: ::::*:*********.:** ****** .:*  **. ****:*:

GzmB-Mouse      SWIKKTMKSS    247
GzmB-Rat        SWIKKTMKKS    248
GzmB-Human      HWIKKTMKRY    247
GzmB-Monkey     RWIKKTMKRH    247
GzmB-Pig        PWIKKIMKSL    247
GzmB-Bovine     PWIKKTMKSL    246
                 **** **
```

C.

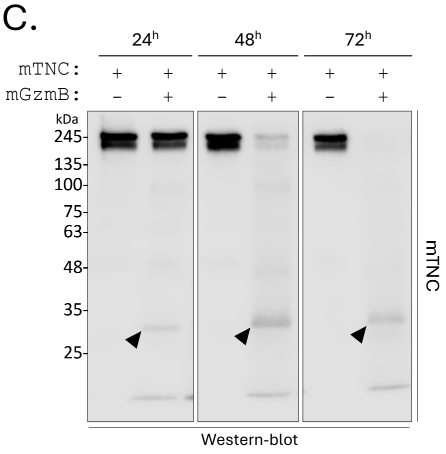

Supplementary figure 2: (**A**) Identify matrix between amino-acid sequences of GzmB from different species, represented as percentage of identity (same amino-acid at the same position). Identity percentage between human and mouse GzmB is indicated in bold/red. (**B**) Amino-acid sequence alignment between GzmB from different species. Residues labelled with (*) are identical, residues labelled with (:) are highly conserved and residues labelled with (.) are poorly conserved. Amino-acids from the catalytic triad (His^{59}-Asp^{103}-Ser^{198}) are identified in bold and are highlighted in yellow. (**C**) 500 ng of lab-generated rmTNC was incubated with (or without) 50 nM rmGzmB for 24h, 48, or 72h at 37°C and analyzed by western blot probing for mTNC. Arrowheads are pointing at the 30 kDa fragments of mTNC generated by mGzmB digestion.

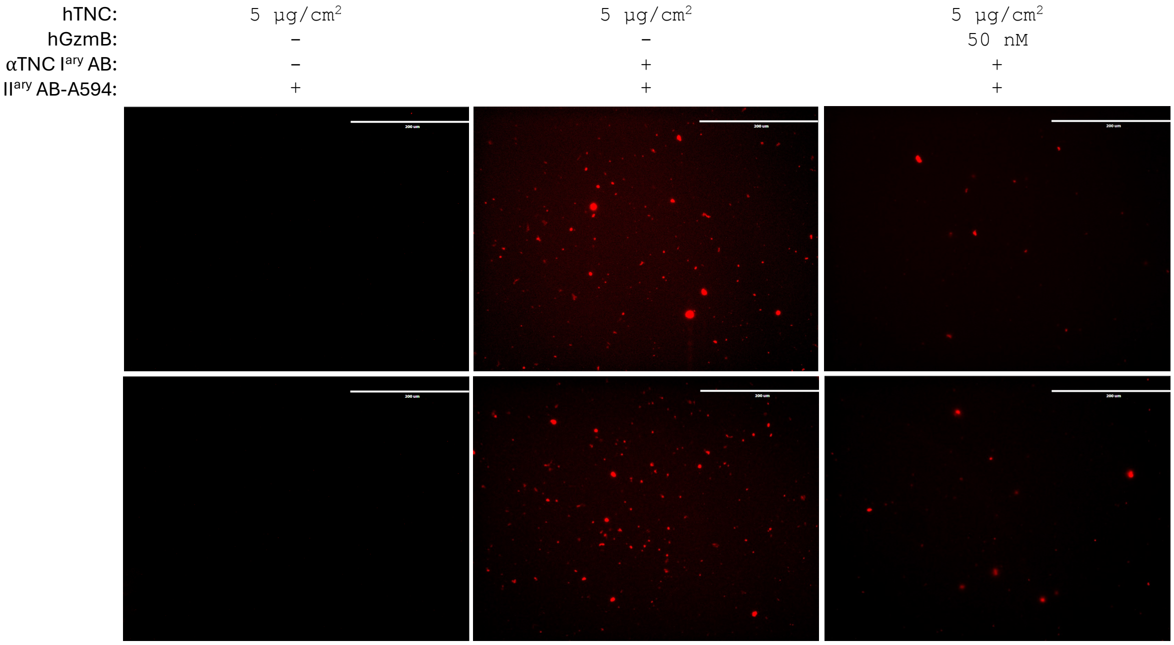

Supplementary figure 3: Immunofluorescence staining performed against immobilized rhTNC non-digested or digested with 50 nM GzmB for 24h. Bars, 200 µm.

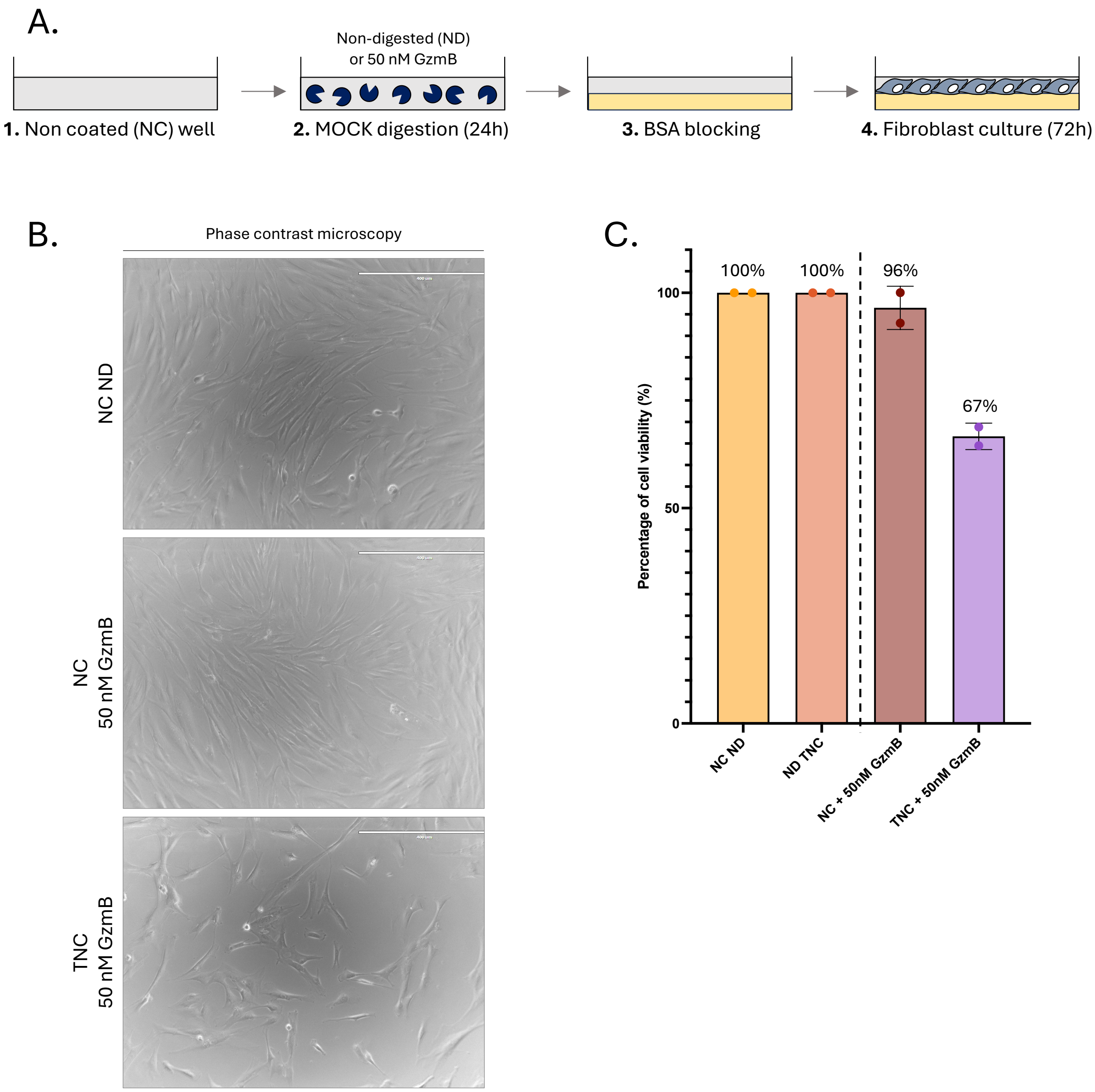

Supplementary figure 4: (**A**) Schematic representation of the experimental procedure used to investigate the direct impact of GzmB on fibroblast behaviours. (**B**) Phase-contrast microscopy performed on primary human dermal fibroblasts cultured for 72 h onto non-coated (NC) and non-digested (ND) wells, NC wells digested with rhGzmB, or TNC coated wells digested with rhGzmB as indicated in (**A**). Bars, 400 µm. (**C**) Percentage of cell viability obtained by MTS assay performed on primary human dermal fibroblasts cultured

for 72 h onto NC/ND wells, NC wells digested with rhGzmB, wells coated with ND rhTNC, or wells coated with rhTNC and digested with rhGzmB. Results were normalized compared to their respective control condition and are represented as means ± SD from 2 independent experiments. NC = non-coated. ND = non-digested.

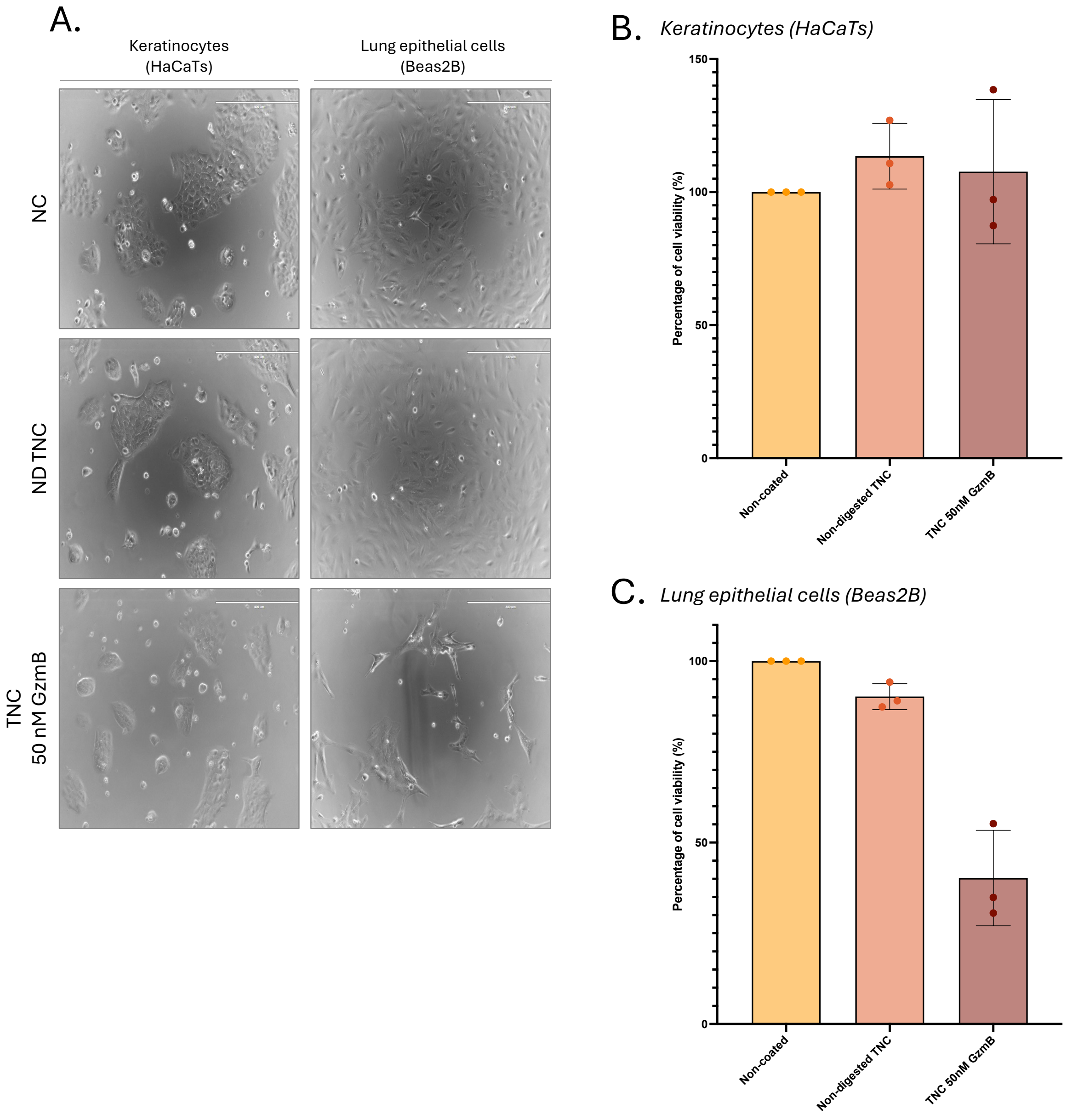

Supplementary figure 5: (**A**) Phase-contrast microscopy performed on keratinocytes (HaCaTs, left panel) or lung epithelial cells (Beas2B, right panel) cultured for 72 h onto non-coated (NC) wells, wells coated with non-digested (ND) rhTNC, or wells coated with rhTNC and digested for 24h with 50 nM rhGzmB. Bars, 400 µm. (**B and C**) Percentage of cell viability obtained by MTS assay performed on HaCaTs (**B**) or Beas2B (**C**) cultured for 72 h onto non-coated (NC) wells, wells coated with non-digested (ND) rhTNC, or wells

coated with rhTNC and digested for 24h with 50 nM rhGzmB. Results were normalized compared to the NC condition and are represented as means ± SD from 3 independent experiments. NC = non-coated. ND = non-digested.

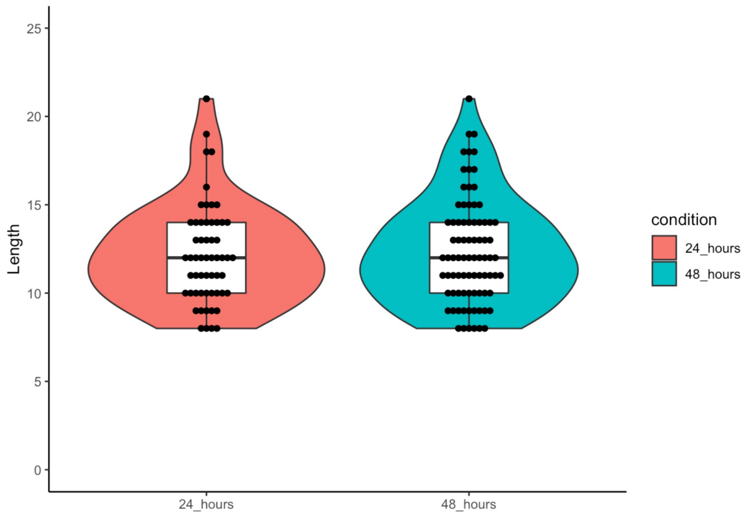

Supplementary figure 6: Violin plots showing the distribution of peptide lengths observed after rhTNC digestion by rhGzmB for 24h (red) or 48h (blue). The overlaying box plots were in the style of Tukey: the box boundaries represent Q1 and Q3 while the line within the box represents the median; whiskers extend from the upper and lower quartiles to the maximum and minimum values, respectively, with the outliers excluded. Outliers were defined as 1.5 × IQR of Q1 or Q3 and are displayed as points beyond the minimum and maximum values, respectively.

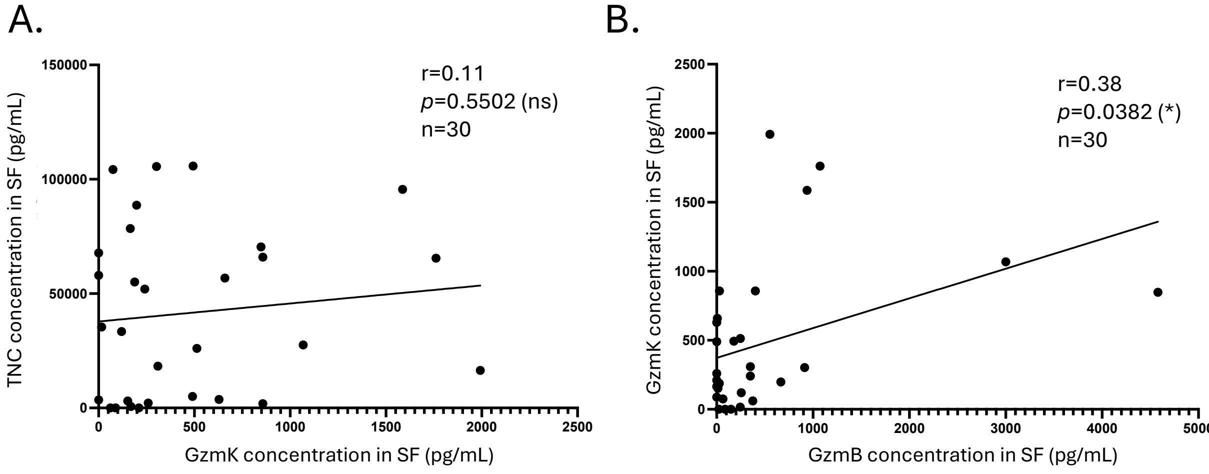

Supplementary figure 7: (A and B) Correlation (Spearman rank-order) between TNC and GzmK levels (**A**) or between GzmK and GzmB levels (**B**) in HC, OA, RA and IA patient's synovial fluids measured by ELISA. ns = not significant, *=p<0.05.
