# Supplementary material for: Granzyme B cleaves tenascin-C to release its C-terminal domain in rheumatoid arthritis
Source: JCI Insight. 2024 Oct 30;9(23):e181935. doi: 10.1172/jci.insight.181935 (PMC11623945; doi:10.1172/jci.insight.181935)

Full unedited membranes for figure 2A

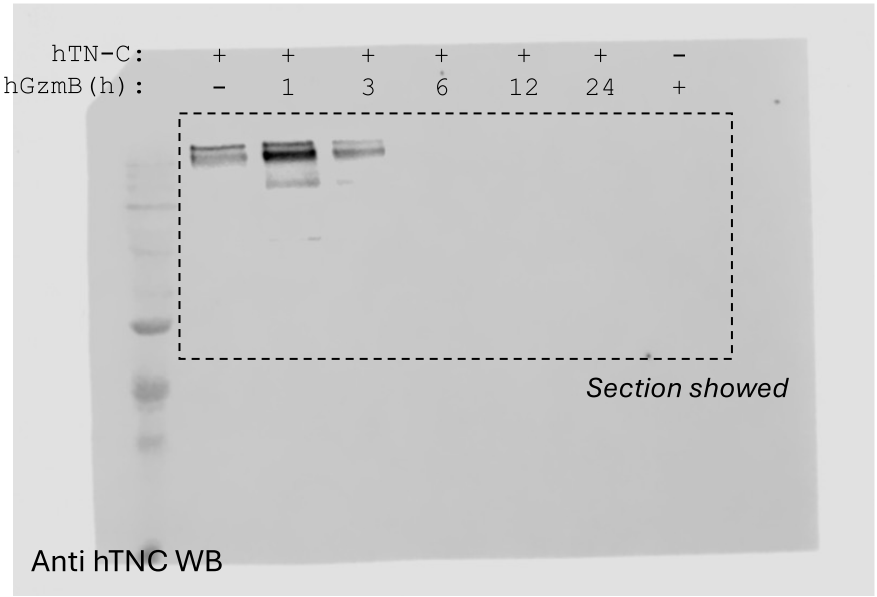

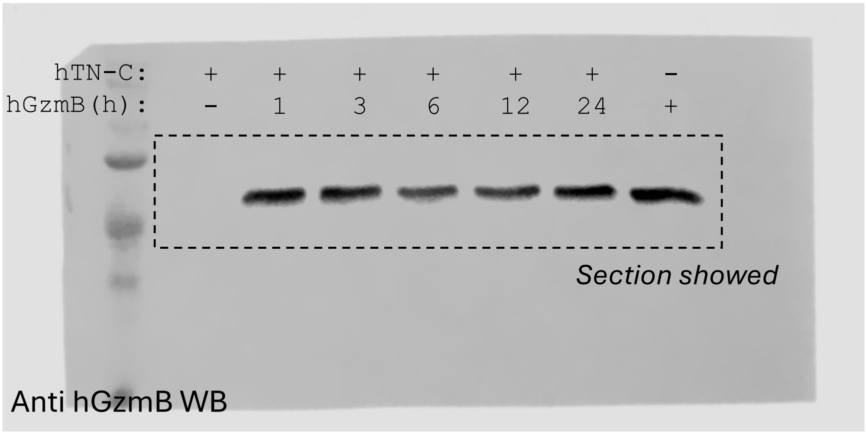

Full unedited membranes for figure 2B

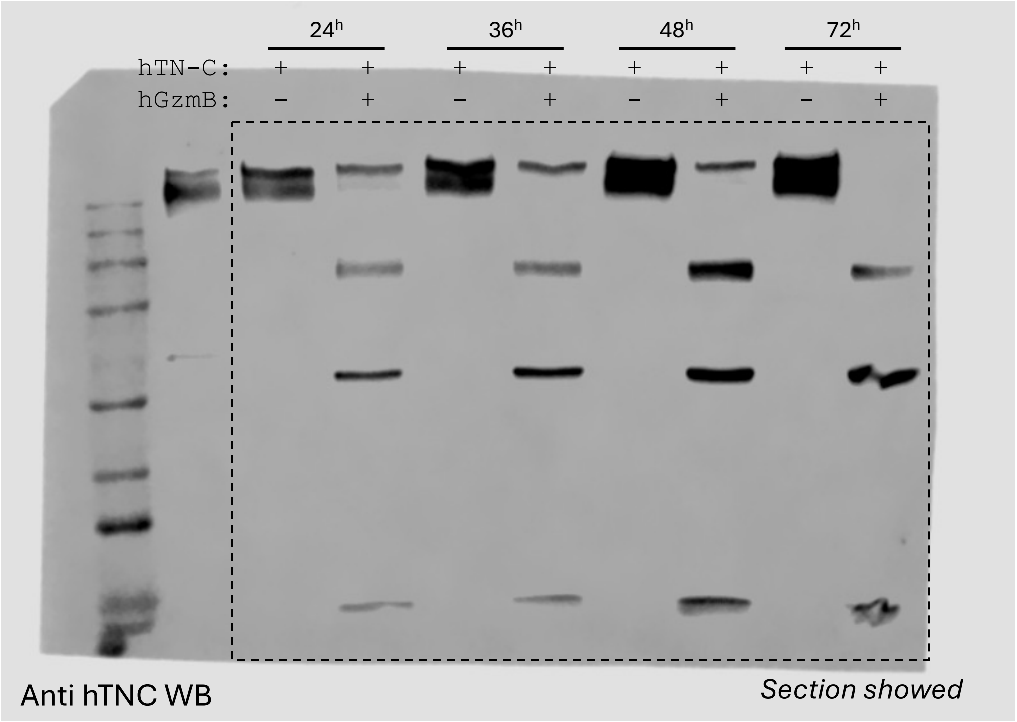

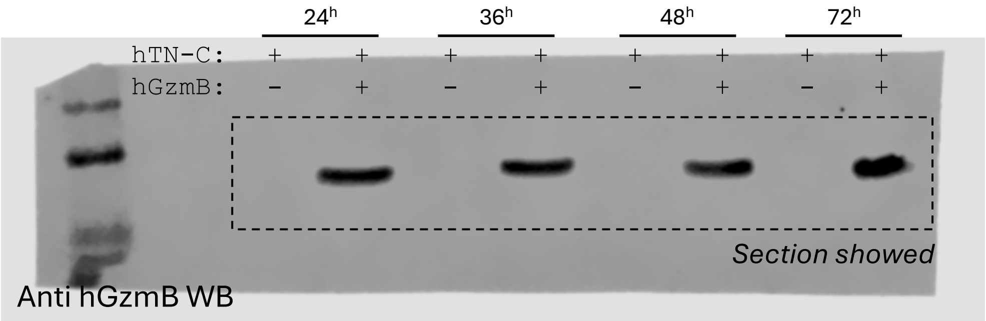

Full unedited membranes for figure 2D

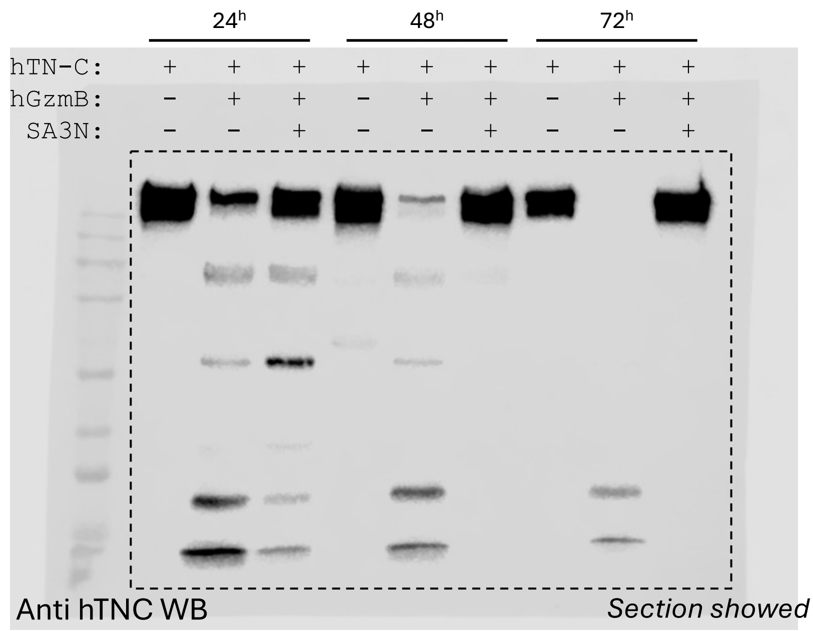

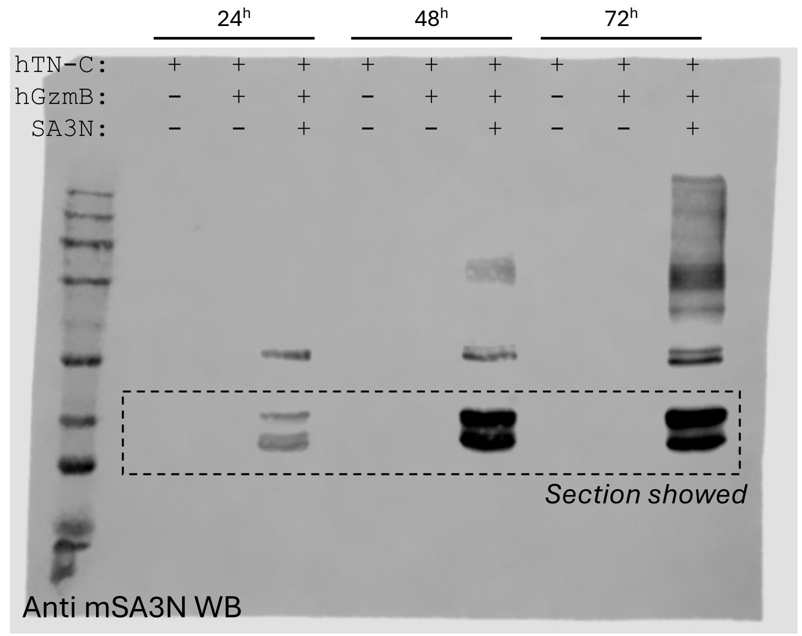

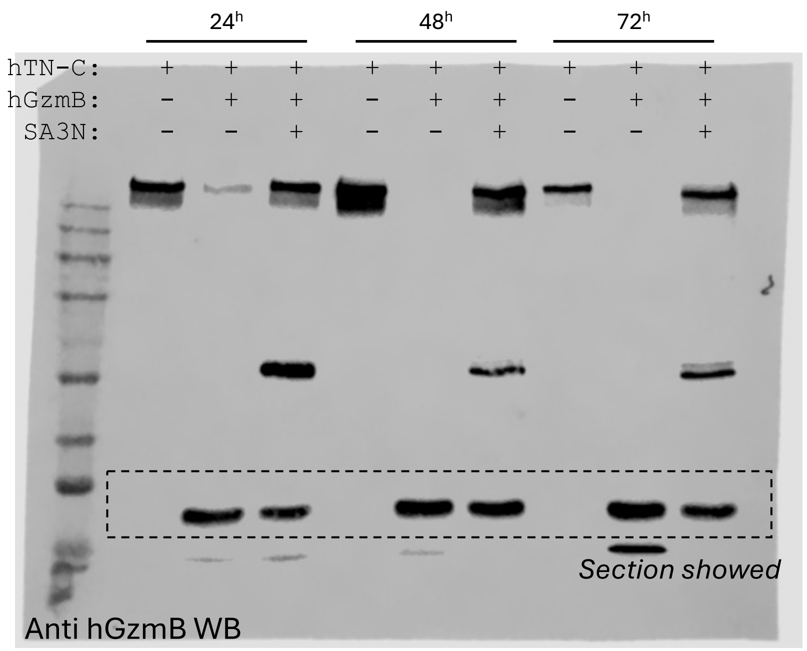

Full unedited membrane for figure 3B

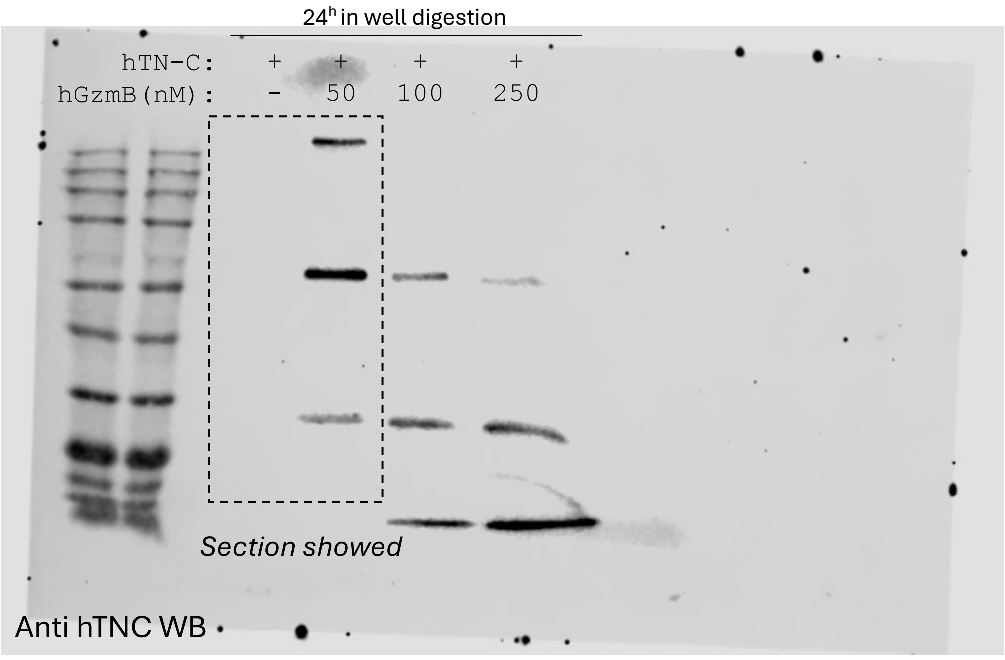

Full unedited membranes for figure 5D (RA Synovial fluids)

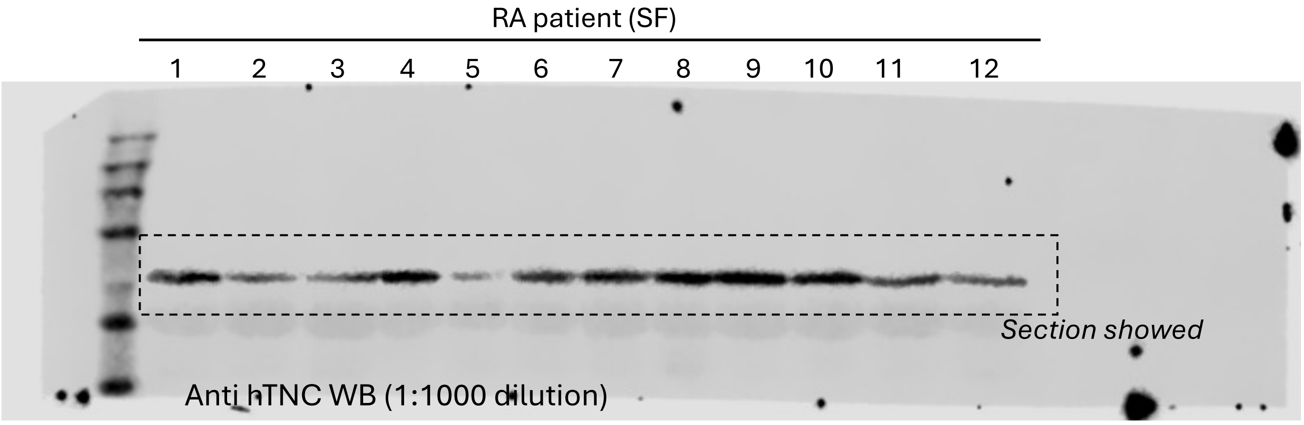

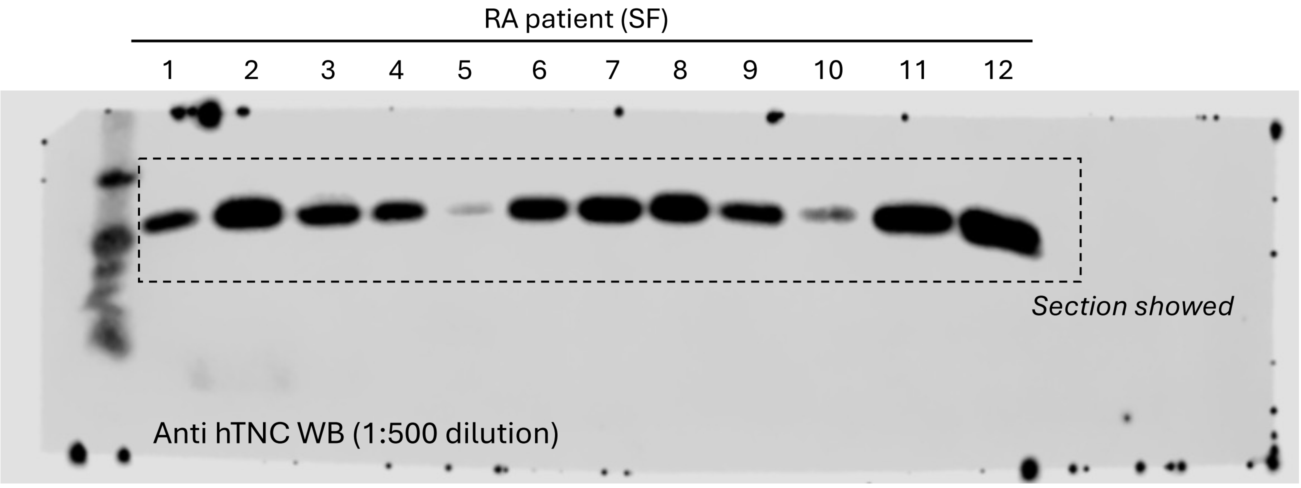

Full unedited membranes for figure 6A - left panel (50nM GzmK)

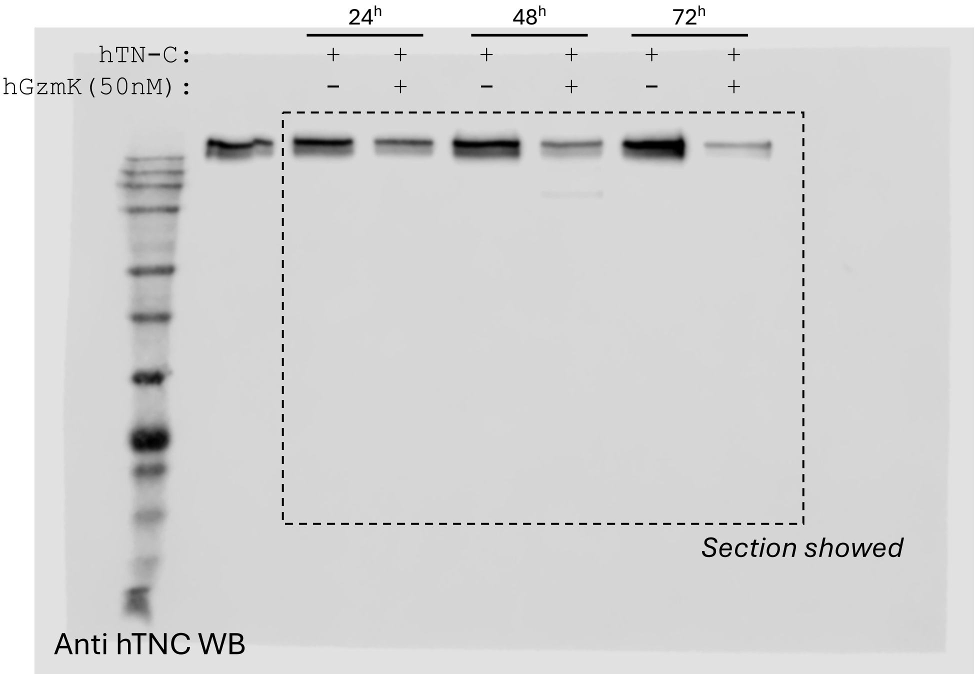

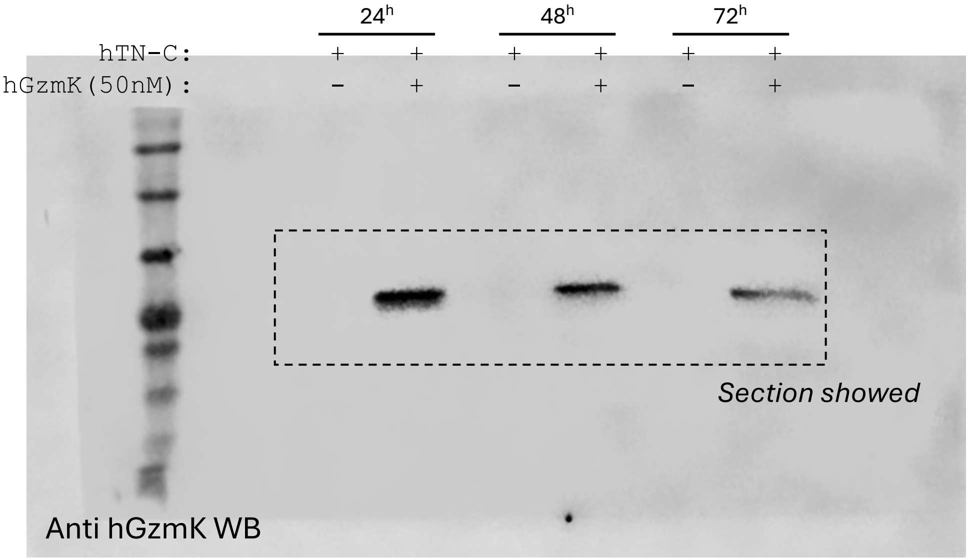

Full unedited membranes for figure 6A - right panel (100nM GzmK)

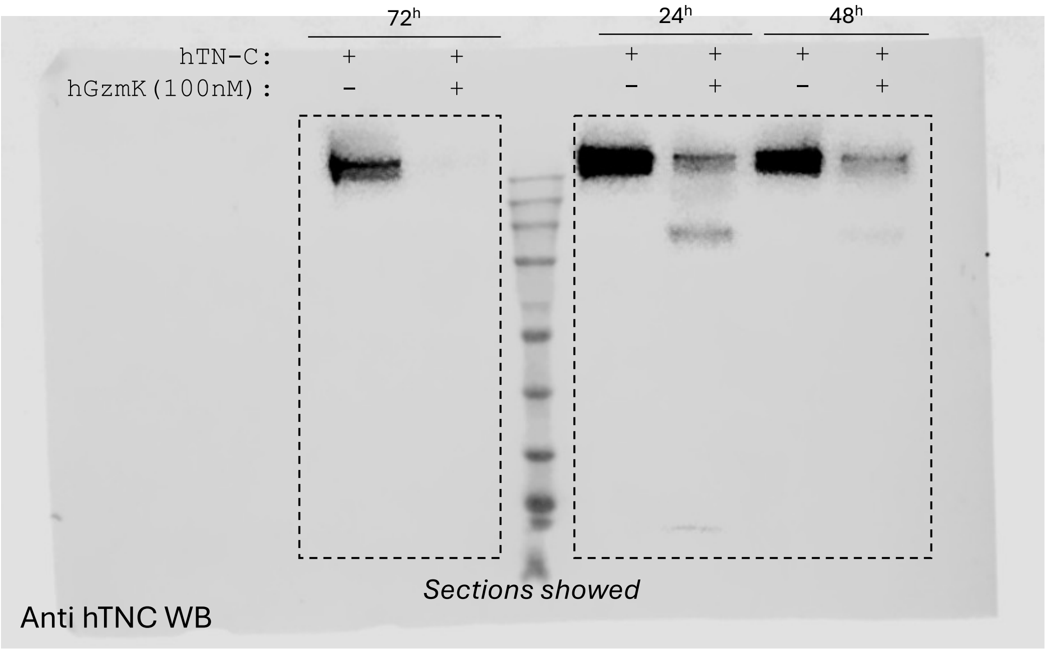

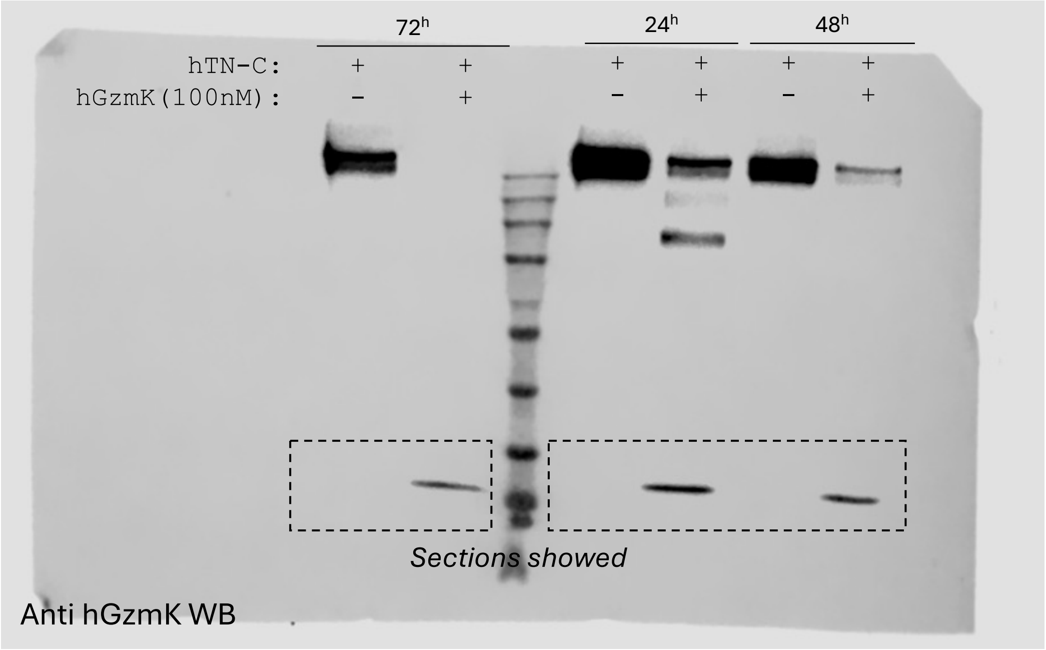

Supplement: Unedited blot and gel images [file jciinsight-9-181935-s011.pdf]
